# CAG-Repeat RNA Hairpin Folding and Recruitment to Nuclear Speckles with a Pivotal Role of ATP as a Cosolute

**DOI:** 10.1021/jacs.2c13653

**Published:** 2023-04-16

**Authors:** Alexander Hautke, Arthur Voronin, Fathia Idiris, Anton Riel, Felix Lindner, Amandine Lelièvre-Büttner, Jikang Zhu, Bettina Appel, Edoardo Fatti, Karsten Weis, Sabine Müller, Alexander Schug, Simon Ebbinghaus

**Affiliations:** † Institut für Physikalische und Theoretische Chemie, TU Braunschweig, Rebenring 56, Braunschweig 38106, Germany; ‡ Steinbuch Centre for Computing, Karlsruher Institut für Technologie, Herrmann-von-Helmholtz-Platz 1, Eggenstein-Leopoldshafen 76344, Germany; § Institut für Biochemie, Universität Greifswald, Felix-Hausdorff-Straße 4, Greifswald 17487, Germany; ∥ Institut für Biochemie, ETH Zürich, Otto-Stern-Weg 3, Zürich 8093, Switzerland; ⊥ Jülich Supercomputing Centre, Forschungszentrum Jülich, Wilhelm-Johnen-Str., Jülich 52452, Germany; # Faculty of Biology, University of Duisburg/Essen, Essen 45141, Germany

## Abstract

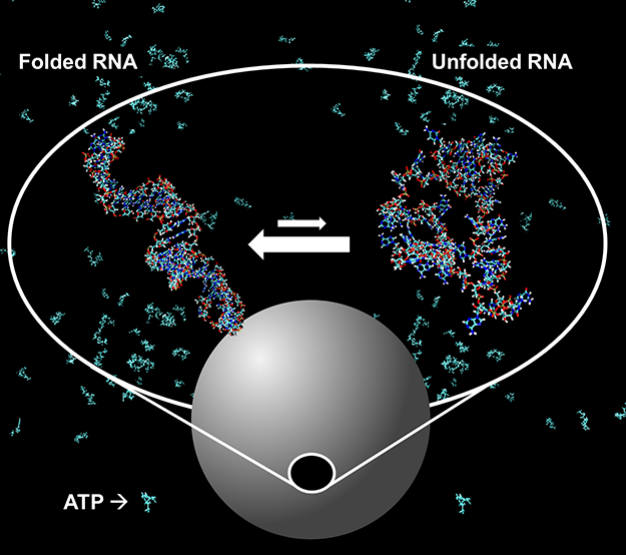

A hallmark of Huntington’s disease (HD) is a prolonged polyglutamine sequence in the huntingtin protein and, correspondingly, an expanded cytosine, adenine, and guanine (CAG) triplet repeat region in the mRNA. A majority of studies investigating disease pathology were concerned with toxic huntingtin protein, but the mRNA moved into focus due to its recruitment to RNA foci and emerging novel therapeutic approaches targeting the mRNA. A hallmark of CAG-RNA is that it forms a stable hairpin in vitro which seems to be crucial for specific protein interactions. Using in-cell folding experiments, we show that the CAG-RNA is largely destabilized in cells compared to dilute buffer solutions but remains folded in the cytoplasm and nucleus. Surprisingly, we found the same folding stability in the nucleoplasm and in nuclear speckles under physiological conditions suggesting that CAG-RNA does not undergo a conformational transition upon recruitment to the nuclear speckles. We found that the metabolite adenosine triphosphate (ATP) plays a crucial role in promoting unfolding, enabling its recruitment to nuclear speckles and preserving its mobility. Using in vitro experiments and molecular dynamics simulations, we found that the ATP effects can be attributed to a direct interaction of ATP with the nucleobases of the CAG-RNA rather than ATP acting as “a fuel” for helicase activity. ATP-driven changes in CAG-RNA homeostasis could be disease-relevant since mitochondrial function is affected in HD disease progression leading to a decline in cellular ATP levels.

## Introduction

Huntington’s disease (HD) belongs to a group of nine neurodegenerative diseases, the so-called polyglutamine diseases, that are caused by the expansion of cytosine, adenine, and guanine (CAG) trinucleotide repeats located in the translated regions of functionally unrelated genes. Yet, they all have a number of shared molecular properties and symptoms, including their mRNAs being capable of forming ribonuclear foci that sequester proteins such as muscleblind protein 1 (MBNL1), leading to aberrant RNA splicing.^1^ In HD patients, one huntingtin (HTT) allele is expanded beyond the pathogenic threshold of 36 repeats,^2^ with the repeat length being inversely correlated with the age of onset of symptoms including involuntary movements and dementia.^2^ Both the mRNA transcript and the protein translated from the mutant allele exert pathogenic effects above this threshold.^1^ As such, the HTT mRNA appears to be a promising target for therapeutic intervention, e.g., by RNA interference.^3,4^ Alternatively, small molecules were reported that rescue disease-relevant features of myotonic dystrophies 1^5^ and 2^6^ and amyotrophic lateral sclerosis (ALS)/frontotemporal dementia (FTD)^7^ by either preventing RNA-binding proteins from interacting with their pathology-related target or degrading them by recruiting RNase L.

A key to such developments is a comprehensive understanding of the conformation and cellular distribution of HTT mRNA. In vitro experiments and simulations showed that CAG-repeat RNAs form a stable hairpin with increasing folding stability for higher repeat numbers due to the repetitive stable pairing of G and C nucleotides.^8^ In the context of HTT exon 1, base pairing with the CAG flanking sequences supports an ensemble of hairpin conformations not limited to the CAG-repeat region.^9^ Remarkably, extended CAG-RNA is retained in the nucleus within RNA foci inside nuclear speckles, leading to the sequestration of different RNA binding proteins.^1,3,10^ The binding affinity depends on the CAG-repeat length and, thus, the hairpin folding stability^11^ and is associated with disease pathology.^1,10,11^

Nuclear speckles contain high quantities of pre-mRNA splicing factors, transcription factors, and 3′-end RNA processing factors.^12,13^ They are classified as membraneless phase-separated organelles that exhibit liquid-like behavior and could play a functional role in the spatial organization of RNA splicing.^14^ Jain and Vale showed that CAG-repeat mRNA sequestered in nuclear speckles is mobile.^15^ In vitro experiments further revealed that CAG-repeat RNA itself forms clusters in condensates at concentrations as low as 25 nM.^15^ However, in these assemblies, the RNA does not retain dynamic behavior, and the condensates are thus classified as gels. Simulations suggest that this self-association behavior can be attributed to intermolecular base pairing of (partially) unfolded CAG hairpins.^16^ To explain the high mobility of CAG-RNA in nuclear speckles compared to its self-association in gels, Jain and Vale hypothesized that helicases in the nucleoplasm could remodel CAG-repeat RNA base pairing.^15^

The fact that nuclear speckles have a liquid-like character and the ability of CAG triplet repeat RNA to form gels suggest compelling research questions regarding CAG-RNA homeostasis: What is the molecular driving force of CAG-RNA-condensate association? In which conformation is the CAG-RNA sequestered? Do aberrant CAG entanglements/aggregates form preferentially in the nucleoplasm or in condensates? Is the sequestration of potentially toxic RNA a cytoprotective mechanism, e.g., by preventing non-native binding events in either of the environments? If this mechanism is exhausted, could nuclear speckles undergo pathogenic aberrant phase transitions?

In this study, we explore these questions by investigating the conformational equilibrium and mobility of CAG-RNA in different cellular environments and conditions.

## Results

### Sequestration of CAG-RNAs in Nuclear Speckles

**Figure 1:**
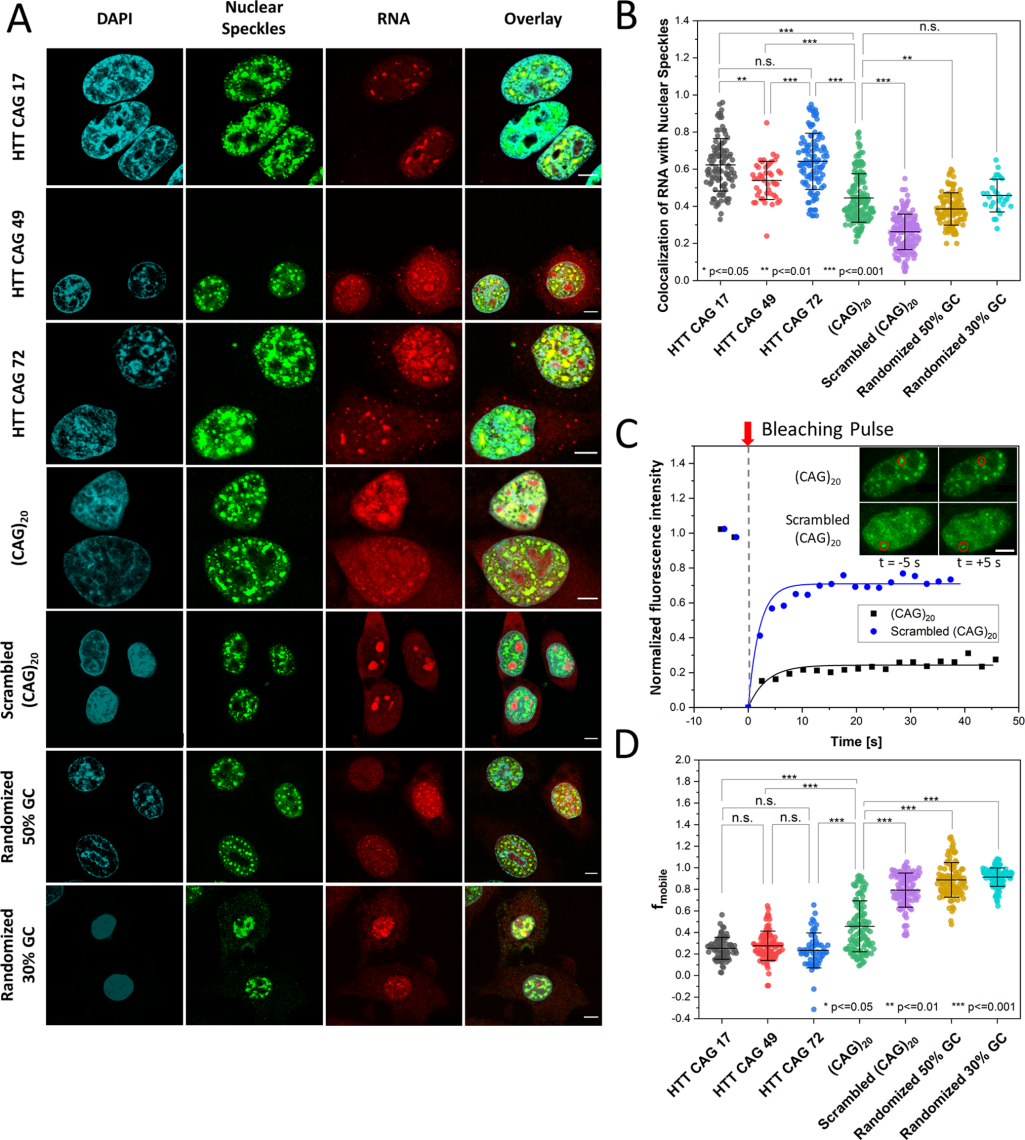
CAG-RNA colocalization with nuclear speckles and mobility in RNA foci. (A) Sample images showing the colocalization of CAG-RNAs (Alexa594) and nuclear speckles (SC35 nuclear speckle marker/Alexa488). Scale bars: 5 μm. (B) Statistical analysis of colocalization for the respective CAG-RNAs. (C) Normalized fluorescence intensity plotted against time for sample FRAP experiments with (CAG)_20_ and scrambled (CAG)_20_. Inset: pre- and post-bleach images of (CAG)_20_ (top row) and scrambled (CAG)_20_ (bottom row) foci in the nuclei of HeLa cells. The bleaching region is marked by a red circle. Scale bar: 5 μm. (D) Mobile fractions of CAG-RNAs in nuclear speckles (CAG-repeat RNAs) and other foci [scrambled (CAG)_20_, randomized 50% GC, and randomized 30% GC]. Statistical analyses shown in (B,D) were performed by a one-way ANOVA and post-hoc Tukey test (for sample sizes, see Table S1). Error bars show S.D. calculated by Gaussian error propagation (see the Supporting Information Materials and Methods section).

First, we investigated how the length, sequence, and flanking regions of the CAG repeats determine recruitment to nuclear speckles and how these properties determine mobility. Therefore, we microinjected fluorophore-labeled (CAG)_20_ RNA into HeLa cells (see the Supporting Information Materials and Methods section for details) at a stock solution concentration of 100 μM (if not stated otherwise), yielding a cellular concentration of approximately 1.4 μM that largely exceeds the endogenous CAG-repeat RNA concentration.^3,17^ Colocalization with nuclear speckles was determined using the SC35 nuclear speckle marker (Figure 1A). We compared (CAG)_20_ RNA to a scrambled sequence [scrambled (CAG)_20_] and to randomized sequences of the same length with a 30 and 50% GC content, respectively. We also included HTT exon 1 sequences with 17, 49, and 72 CAG repeats. These were employed at lower stock solution concentrations (100–200 ng/μL) as yielded after in vitro transcription and stochastic fluorescent labeling. While (CAG)_20_ RNA and all HTT exon 1 constructs were found to be predominantly localized in nuclear speckles, the randomized RNAs formed distinct small foci inside the nucleoplasm, and the scrambled RNA was mainly found in the nucleoli and the cytosol, with some small foci still being observed in the nucleoplasm [sample images: Figure 1A,C (inset) and evaluation of the focus size, number, and intensity: Figure S1].

We further analyzed the extent of colocalization of the RNAs with nuclear speckles by an object-based approach measuring the overlap of RNA foci and nuclear speckles using CellProfiler^18−21^ (see the Supporting Information Materials and Methods section for details). (CAG)_20_ RNA showed a significantly higher colocalization share with nuclear speckles (45 ± 13%) compared to the scrambled and randomized sequences [scrambled (CAG)_20_: 26 ± 10%; randomized 50% GC: 39 ± 9%; and randomized 30% GC: 46 ± 9%] (Figure 1B). Colocalization of randomized 50% GC and randomized 30% GC was mainly caused by arbitrary overlaps between small RNA foci and nuclear speckles (Figure 1A). The highest colocalization was found for HTT exon 1 sequences including both the CAG tract and its physiological flanking sequences (HTT CAG 17: 62 ± 14%; HTT CAG 49: 54 ± 10%; and HTT CAG 72: 64 ± 15%). We did not find a significant difference between HTT exon 1 RNAs with physiological (HTT CAG 17) and pathologically expanded CAG repeats (HTT CAG 49 and HTT CAG 72).

To further corroborate these results, we conducted fluorescence recovery after photobleaching (FRAP) measurements in the different foci (Figure 1C). We found that the HTT exon 1 constructs exhibited the lowest mobility as shown by lowest mobile fraction (*f*_mobile_) values. *f*_mobile_ is determined by the fraction of fluorescence that recovers throughout the course of a FRAP experiment (Figure 1C). This finding is in line with a strong association of the RNA with nuclear speckles. Again, no significant change in mobility between the different CAG repeat length extensions (HTT CAG 17: 25 ± 10%; CAG 49: 28 ± 14%; and CAG 72: 23 ± 16%) was found (Figure 1D). The (CAG)_20_ RNA showed slightly higher mobility compared to the HTT exon 1 RNAs [(CAG)_20_: 46 ± 24%], and the randomized and scrambled sequences of equal length showed significantly increased mobility [scrambled (CAG)_20_: 79 ± 16%; randomized 50% GC: 89 ± 16%; and randomized 30% GC: 91 ± 9%]. Microinjections at different (CAG)_20_ stock concentrations (10, 100, and 300 μM) showed that the mobility measurements were independent of the cellular concentration (Figure S2).

In comparison to earlier studies by Jain and Vale,^15^ who measured a mobile fraction of 83 ± 13% for a (CAG)_49_ sequence in nuclear speckles, the CAG-RNAs investigated here showed smaller mobile fractions [e.g., (CAG)_20_: 46 ± 24%]. In their earlier study, Jain and Vale conjugated the CAG-RNA with 12 MS2 hairpin loops binding to yellow fluorescent protein-labeled MS2-binding protein. In comparison to the Alexa Fluor dyes used here, their bulky detection system could potentially affect the phase behavior, e.g., by diminishing intermolecular CAG interactions in the condensate.

In summary, the colocalization and FRAP studies both show that an increased association of CAG-RNA sequences with nuclear speckles is accompanied by a decrease in mobility suggesting stronger association with the condensate. Association and interaction with nuclear speckles are specific to the CAG repetition motive and enhanced by the flanking sequences of HTT exon 1. For the here-studied HTT exon 1 constructs, these properties are independent of CAG-repeat length.

### (CAG)_20_ RNA Hairpins Are Stable but Destabilized in Cells

Based on these results, we proceeded to study (CAG)_20_ RNA as a model system to analyze its conformational stability as a function of subcellular localization. We used fast relaxation imaging (FReI) that allows us to measure the folding kinetics and thermodynamics of biomolecules in cells with high spatio-temporal resolution by combining fluorescence resonance energy transfer (FRET) microscopy with fast temperature jumps induced by an infrared (IR) laser.^22−24^ The technique was previously used to study folding and phase behavior of superoxide dismutase 1,^25,26^ conformational dynamics and aggregation of Huntingtin exon 1,^27,28^ or the folding stability of 4U-RNA in the nucleus and cytoplasm of cells.^29^

**Figure 2:**
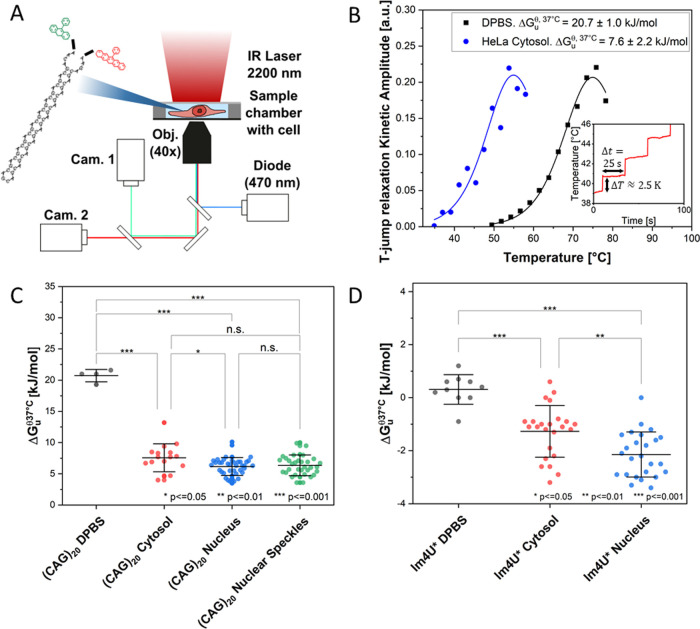
Folding stability of (CAG)_20_ measured by FReI. (A) Schematic of the FReI microscope and (CAG)_20_ RNA (see Figure S4A for details). Thermal unfolding upon temperature jumps [induced by an IR laser (λ = 2200 nm)] is monitored by FRET [(Alexa Fluor 488/594); donor excitation: λ = 470 nm]. (B) Kinetic relaxation amplitudes measured at different temperatures and fitted by the Girdhar model.^35^ Inset: customized temperature jump protocol calibrated using the temperature-dependent fluorescence of Rhodamine B.^38−40^ (C,D) Δ*G*_u_^θ,37^°^C^ of (CAG)_20_ (C) and lm4U* (D) RNA measured in DPBS and HeLa cell cytosol, nuclei, and nuclear speckles. Statistical analyses were performed by a one-way ANOVA and post-hoc Tukey test (for sample sizes, see Table S1). Error bars show S.D. calculated by Gaussian error propagation (see the Supporting Information Materials and Methods section).

In preliminary work,^30^ we prepared (CAG)_20_ RNA for FReI experiments by terminal FRET labeling using Alexa Fluor 488 at the 5′-end and Alexa Fluor 594 at the 3′-end. Dyes were covalently connected to the RNA backbone via C6 linkers and by alkyne–azide click chemistry (5′-end) or amine coupling to a carbonic acid N-hydroxy succinimide ester (3′-end) (Figure 2A). We characterized the labeled RNA in vitro and established a tailored protocol to measure its folding stability by fast consecutive temperature jumps (Figure 2B, inset), recording the temperature-dependent unfolding kinetics within 8 min in a single living cell with every respective temperature jump lasting 25 s.^30^ We found that the (CAG)_20_ RNA forms stable hairpins in Dulbecco’s phosphate buffered saline (DPBS; pH 7.0–7.3, 2.7 mM KCl, 1.5 mM KH_2_PO_4_, 140 mM NaCl, 8 mM Na_2_HPO_4_) buffer solution (*T*_m_ = 349 ± 2 K, Δ*G*_u_^θ,37^°^C^ = 20.7 ± 1.0 kJ/mol ),^30^ which was in agreement with previous studies.^3,31^ However, the folding stability was significantly decreased in the presence of cosolutes due to various factors such as transient chemical interactions^30,32,33^ or a decrease in water activity,^30,33,34^ leading to the question whether (CAG)_20_ RNA remains folded in the densely crowded cell or upon sequestration in nuclear speckles.

We therefore microinjected (CAG)_20_ RNA into HeLa cells and measured its folding stability by FReI (Figure 2A). Analysis of the temperature-dependent unfolding kinetics (Figure 2B) allowed us to calculate the melting temperature *T*_m_, first-order cooperativity parameter *g*^(1)^ (the slope at the inflection point of a thermal melting curve), and the standard free energy of unfolding at 37 °C: Δ*G*_u_^θ,37^°^C^ = (310 K – *T*_m_)·*g*^(1)^.^35^ Δ*G*_u_^θ,37^°^C^ was used to calculate the equilibrium constant *K* and folded and unfolded fractions of the RNA at 37 °C (*K*^37^°^C^, *f*_folded_^37^°^C^, = *f*_unfolded_^37^°^C^) (see the Supporting Information Materials and Methods section for details) with up to 1.5–2 μm of spatial resolution in live cells.
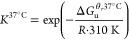

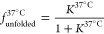

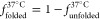


The model is based on the two-state assumption which is supported by single-molecule FRET measurements of (CAG)_20_ RNA in DPBS under native and destabilizing conditions (Figure S3). Instead of the commonly used sigmoidal fitting procedure which requires fully resolved folded and unfolded state baselines, we used a previously established a kinetic method to analyze in-cell melting curves with a limited accessible temperature range.^35^ This method allows for a thermodynamic analysis with only a few data points recorded beyond *T*_m_ (Figure 2B).

The results are summarized in Tables S2 and S3, and Δ*G*_u_^θ,37^°^C^ values are compared in Figure 2C,D. The analysis showed that (CAG)_20_ RNA was largely destabilized in the cellular environment compared to dilute DPBS buffer solution [Δ*G*_u_^θ,37^°^C^(DPBS) = 20.7 ± 1.0 kJ/mol^30^ and Δ*G*_u_^θ,37^°^C^(cytosol) = 6.8 ± 2.1 kJ/mol]. Despite the major destabilization, it is important to note that the RNA was still mostly folded under physiological conditions (Δ*G*_u_^θ,37^°^C^(cytosol) = 6.8 ± 2.1 kJ/mol, meaning that only 6.7% of the RNA was unfolded at 37 °C). The decrease in Δ*G*_u_^θ,37^°^C^ was also large compared to that under any in vitro condition measured previously (e.g., Δ*G*_u_^θ,37^°^C^(DPBS + 300 g/L Ficoll 70) = 18.0 ± 1.0 kJ/mol; Δ*G*_u_^θ,37^°^C^(DPBS + 300 g/L ethylene glycol) = 12.0 ± 3.0 kJ/mol; Δ*G*_u_^θ,37^°^C^(HeLa cell lysate) = 13.3 ± 0.5 kJ/mol).^30^ Thus, crowding effects such as the decrease in water activity or non-specific interactions of the RNA and cosolutes could not solely account for the observed decrease in folding stability.

To further interpret these results, a low-melting but stable RNA hairpin, the *Salmonella* fourU RNA thermometer [lm4U*; *denoting the destabilizing mutation (C23U)], was studied for comparison to CAG hairpins. The secondary structure in comparison to that of a CAG hairpin is shown in Figure S4A,B. The RNA is located in the 5′-untranslated region (5′-UTR) of the aggregation–suppression protein and functions as a temperature sensor in free-living microorganisms.^36,37^ lm4U* was shown to fold in a two-state manner in HeLa cells,^29^ and since it is a prokaryotic RNA functioning as a temperature-sensitive control element in free-living organisms, it is not engaged in specific cellular functions in HeLa cells. lm4U* is marginally stable in DPBS at 37 °C (Δ*G*_u_^θ,37^°^C^(DPBS) = 0.3 ± 0.6 kJ/mol) and slightly destabilized in cells Δ*G*_u_^θ,37^°^C^(cytosol) = −1.3 ± 1.0 kJ/mol, Δ*G*_u_^θ,37^°^C^(nucleus) = −2.1 ± 0.8 kJ/mol) (Figure 2D). Compared to (CAG)_20_ RNA, the destabilization in cells is small and is explained by compensating crowding effects. While the loss in water activity and transient chemical interactions destabilize lm4U*, excluded volume effects lead to a counteracting stabilizing effect.^29^

We then analyzed the folding stability in nuclear speckles for (CAG)_20_ RNA [lm4U* does not associate with nuclear speckles under any condition (Figure S5)]. We found a similar Δ*G*_u_^θ,37^°^C^ in nuclear speckles and the bulk nucleoplasm (Δ*G*_u_^θ,37^°^C^(nucleus) = 6.0 ±1.4 kJ/mol; Δ*G*_u_^θ,37^°^C^(nuclear speckles) = 6.4 ± 1.6 kJ/mol (Figure 2C). This shows that (CAG)_20_ RNA associates with nuclear speckles in the folded rather than unfolded hairpin conformation (92.3% of the RNA is folded in nuclear speckles) and that condensate association is not associated with a conformational transition under physiological conditions.

However, we observed that the (CAG)_20_ RNA is enriched in nuclear speckles upon stepwise heating (Figure S6A–C). In particular, at temperatures beyond the melting point where the RNA is mostly unfolded, strong migration to the foci was observed (Figure S6A–C). We found that the association of the RNA with nuclear speckles is partially irreversible as the partitioning coefficient (PC) between the nuclear speckles and surrounding nucleoplasm does not recover its initial value at 37 °C (Figure S6A,B). Since the RNA unfolds reversibly in a two-state manner in the cytoplasm and nucleoplasm and also under various crowded conditions in vitro, it is not expected that irreversibly misfolded CAG entanglements cause this effect. We analyzed single temperature jumps of FReI experiments performed in nuclear speckles at high temperatures (e.g., 55 °C). Despite this high temperature, (CAG)_20_ RNA unfolding showed no indication of self-association due to intermolecular FRET (Figure S7B), which would be expected in this case due to the high ratio of labeled to endogenous CAG-RNA. In fact, FReI is very sensitive to such self-association events upon unfolding.^22,27,28,41^ Measurements of (CAG)_20_ RNA in concentrated adenosine triphosphate (ATP) solutions (15 and 20 mM) led to microscopically visible aggregates, showing that self-association could be indeed detected for the (CAG)_20_ RNA construct (Figure S8). Thus, the data suggest that CAG-RNA entanglements do not form in speckles (or the nucleoplasm), even at high temperatures and at concentrations much higher than those of endogenous CAG-RNA levels.

### ATP Destabilizes (CAG)_20_ RNA and Maintains Its Mobility in Cells

**Figure 3:**
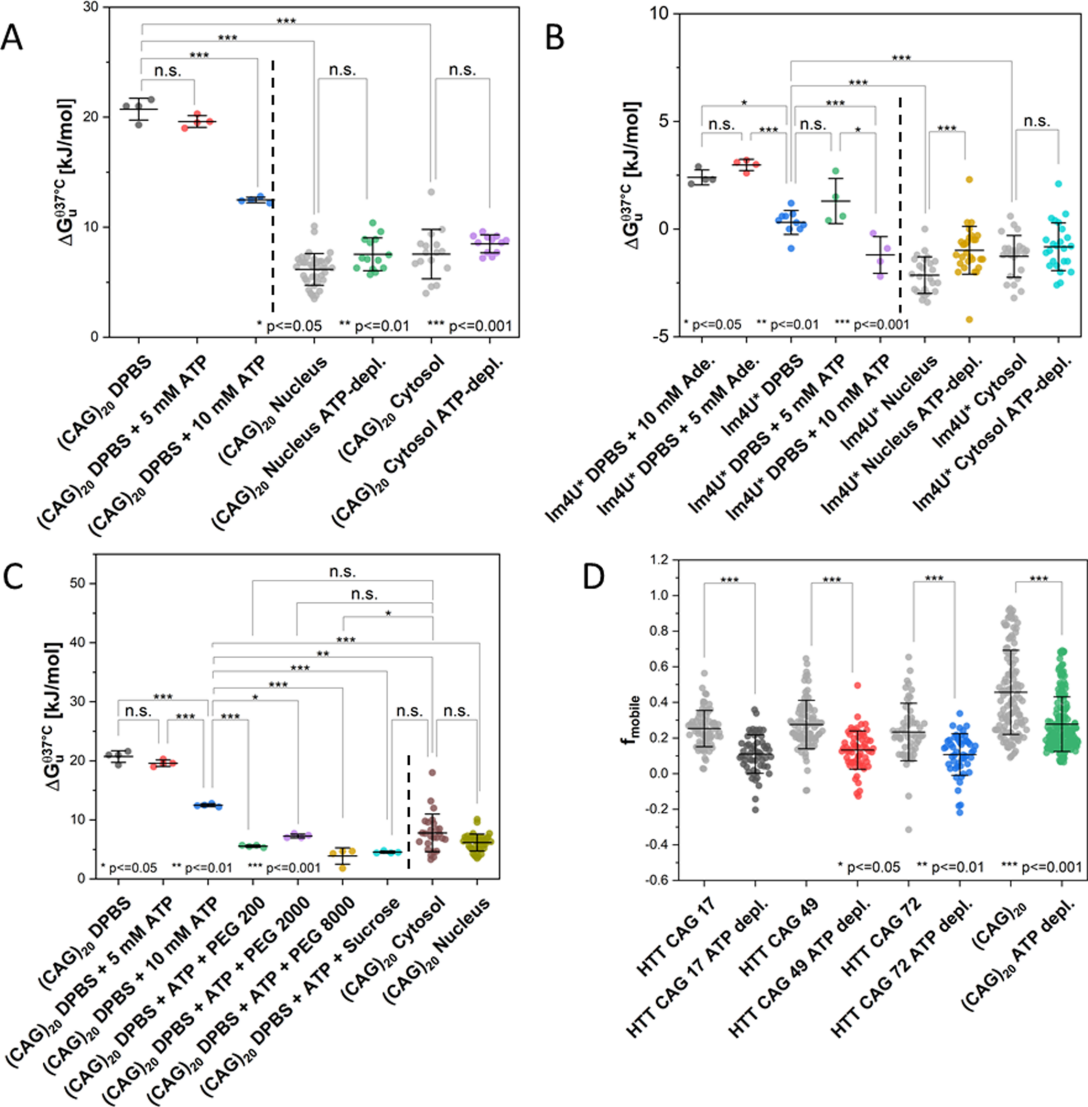
(CAG)_20_ and lm4U* RNA folding stability and mobility as a function of the ATP concentration. A dotted line is used to separate in vitro and in cell data. (A,B) Δ*G*_u_^θ,37^°^C^ measured for (CAG)_20_ and lm4U* in vitro (DPBS) and in cells with and without ATP depletion. (C) Δ*G*_u_^θ,37^°^C^ for (CAG)_20_ in different cosolutes mimicking the cellular environment. If not stated otherwise, the ATP concentration is 10 mM, and cosolute concentrations are 300 g/L. Values from the HeLa cytosol and nucleus are taken from A and shown for comparison. (D) Mobile fraction *f*_mobile_ of (CAG)_20_ and HTT exon 1 RNA in nuclear speckles under normal and ATP-depleted conditions. Data acquired under physiological conditions (Figures 1D and 2C,D) are shown in gray for comparison. Statistical Analysis was performed by a one-way ANOVA and post-hoc Tukey test (for sample sizes, see Table S1). Error bars show S.D. calculated by Gaussian error propagation (see the Supporting Information Materials and Methods section).

Inspired by the previous work of Jain and Vale, who found a profound role of ATP in maintaining the liquid-like properties of CAG repeats in nuclear speckles (e.g., by enabling helicase activity),^15^ we investigated the role of ATP in determining (CAG)_20_ RNA folding stability and mobility in speckles and the nucleoplasm. We first conducted in vitro experiments in the absence of ATP-dependent helicases and other cofactors or binding partners. We found that (CAG)_20_ RNA is increasingly destabilized with rising ATP concentrations (Δ*G*_u_^θ,37^°^C^(DPBS) = 20.7 ± 1.0 kJ/mol; Δ*G*_u_^θ,37^°^C^(DPBS + 5 mM ATP) = 19.6 ± 0.5 kJ/mol; Δ*G*_u_^θ,37^°^C^(DPBS + 7.5 mM ATP) = 17.1 ± 4.9 kJ/mol; and Δ*G*_u_^θ,37^°^C^(DPBS + 10 mM ATP) = 12.5 ± 0.3 kJ/mol) (Figures 3A and S4A). In comparison, lm4U* is slightly stabilized at 5 mM and also largely destabilized at 10 mM (Δ*G*_u_^θ,37^°^C^(DPBS) = 0.3 ± 0.6 kJ/mol; Δ*G*_u_^θ,37^°^C^(DPBS + 5 mM ATP) = 1.3 ± 1.0 kJ/mol; and Δ*G*_u_^θ,37^°^C^(DPBS + 10 mM ATP) = −1.2 ± 0.9 kJ/mol) (Figure 3B). All experiments were performed in magnesium-free DPBS buffer. Thus, an indirect effect of ATP on Δ*G*_u_^θ,37^°^C^ by chelating magnesium ions and removing them from the RNA can be ruled out.

Experiments using adenosine alone showed an increase in folding stability (Δ*G*_u_^θ,37^°^C^(lm4U*; DPBS + 5 mM adenosine) = 3.0 ± 0.3 kJ/mol; Δ*G*_u_^θ,37^°^C^(lm4U*; DPBS + 10 mM adenosine) = 2.4 ± 0.3 kJ/mol; see Figure S4), suggesting that the triphosphate group is required to mediate the destabilization. In accordance with similar observations regarding proteins,^42^ the triphosphate could increase the solubility of ATP, thus facilitating its interaction as a cosolute with the RNA. Furthermore, the extent of destabilization of (CAG)_20_ RNA by ATP is remarkable since similar Δ*G*_u_^θ,37^°^C^ values were only found for small cosolutes like ethylene glycol, PEG 200, or sucrose.^30^ However, this destabilization occurred at significantly lowered water activity and cosolute concentrations that are about 50-fold higher. Neither crowding nor ATP destabilization alone could mimic in-cell folding stability, but remarkably, we found that solutions containing crowding agents [PEG 200, PEG 2000, PEG 8000, and sucrose (300 g/L)] in addition to 10 mM ATP (Figure 3C) could do so. This shows that crowders and ATP both play a role in determining (CAG)_20_ RNA folding stability under cellular conditions. However, further studies are required to define the contribution of ATP in cell-mimicking environments, accounting also for changes, e.g., in pH or ionic strength.

To probe whether changes in the ATP concentration also affect the folding stability of (CAG)_20_ and lm4U* in cells, we conducted ATP depletion experiments, adding final concentrations of 1 mM KCN and 10 mM 2-deoxyglucose to the medium.^43^ ATP depletion was monitored by the ATeam ATP concentration sensor (see Figure S9 and Supporting Information Materials and Methods for further details),^43,44^ and (CAG)_20_ RNA folding stability was assessed at the minimum ATP level. The normalized FRET ratio decreased from 1.0 to 0.6, corresponding to a decrease in the cellular ATP concentration by 2–3 mM.^45^ As ATP levels are estimated to be 2–3 mM in human cells,^46^ it is assumed that ATP is completely depleted from the cells. Indeed, we found that ATP depletion caused a minor stabilization of (CAG)_20_ RNA by ΔΔ*G*_u_^θ,37^°^C^ ≈ 1.5 kJ/mol in both the cytosol and nucleus (Δ*G*_u_^θ,37^°^C^(cytosol, ATP – depl.) = 8.5 ± 0.8 kJ/mol, Δ*G*_u_^θ,37^°^C^(nucleus, ATP – depl.) = 7.5 ± 1.5 kJ/mol) of HeLa cells (Figure 3A). For lm4U*, an even smaller stabilizing effect was observed (ΔΔ*G*_u_^θ,37^°^C^(cytosol) ≈ 0.5 kJ/mol, ΔΔ*G*_u_^θ,37^°^C^(nucleus) ≈ 1.1 kJ/mol) (Figure 3B).

Moreover, ATP depletion led to a significant decrease in mobility (10–20%) of (CAG)_20_ and HTT exon 1 RNA recruited to nuclear speckles [HTT CAG 17: 11 ± 11%; HTT CAG 49: 13 ± 11%; HTT CAG 72: 11 ± 12%; and (CAG)_20_: 28 ± 15%] (Figure 3D). Remarkably, ATP depletion fully prevented further heat-induced recruitment of (CAG)_20_ from the nucleoplasm to the nuclear speckles and completely immobilized RNA that had already been recruited to the nuclear speckles prior to the beginning of the experiment. PCs were independent of temperature (see Figure S6D,E).^15^

These results are in line with the observations by Jain and Vale who found mobile fractions of 83 ± 13% under normal and 23 ± 7% under ATP-depleted conditions. In their study, Jain and Vale suggested that ATP depletion led to a decrease in mobility due to a decline in activity of RNA chaperones with unwinding helicase activity (e.g., from DEAD box family chaperones^15^). From this hypothesis, it can be deduced that unfolding of RNA hairpins, e.g., by RNA helicases should lead to decreased hairpin folding stability. However, since our in vitro results showed that ATP largely decreases CAG hairpin folding stability as a cosolute in the absence of RNA-unwinding helicases, we propose that the measured changes in both folding stability and mobility may be caused by direct interactions of ATP with the RNA.

**Figure 4:**
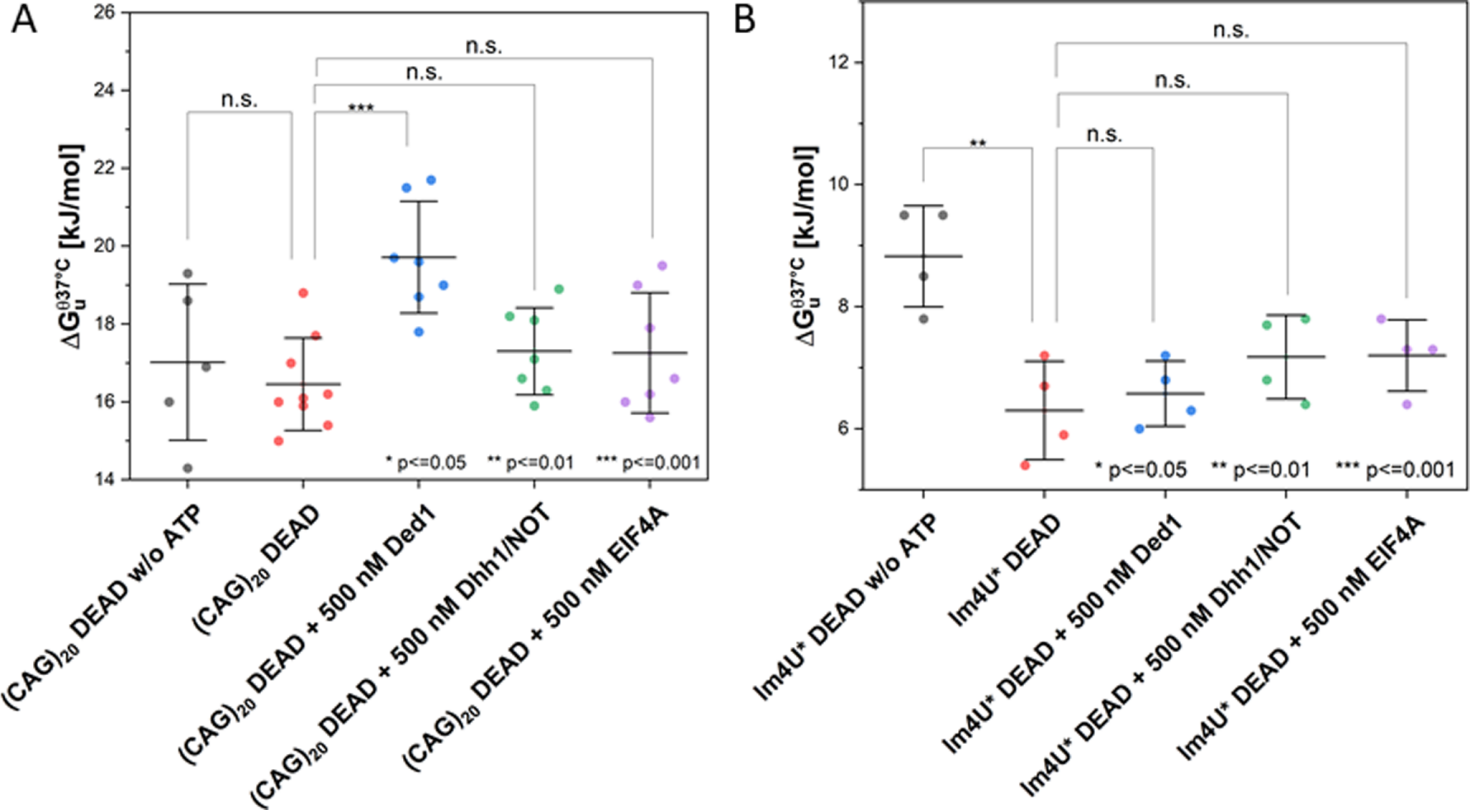
Δ*G*_u_^θ,37^°^C^ for (CAG)_20_ (A) and lm4U* (B) in the presence of DEAD box helicases in DEAD buffer (containing 5 mM ATP if not specified otherwise). Statistical analysis was performed by a one-way ANOVA and post-hoc Tukey test (for sample sizes, see Table S1). Error bars show S.D. calculated by Gaussian error propagation (see the Supporting Information Materials and Methods section).

To further test this hypothesis, we performed in vitro experiments employing three different DEAD box helicases (Ded1, Dhh1, and EIF4A). Indeed, we found that the helicases did not change RNA folding stability compared to the respective buffer solution (Figure 4). This could be rationalized by the known limited RNA helicase capacity of DEAD box chaperones. Their helicase activity decreases with the increasing number and folding stability of base pairs within an RNA duplex as the binding of the protein’s RecA domain to the RNA has to outcompete binding interactions within the RNA. This process becomes ineffective at more than 15 base pairs or even less if the duplex mostly consists of C–G pairs.^47,48^ (CAG)_20_ and lm4U* comprise 18 and 14 base pairs, respectively. In the case of (CAG)_20_, all of these are highly stable C–G pairs. Thus, it can be assumed that DEAD box chaperones do not modulate the folding stability and mobility of CAG repeat hairpins, especially not for the more elongated and stable hairpins linked to the disease. Mobility and folding stability of these RNAs are rather governed by ATP as a cosolute.

### Molecular Dynamics Simulations Reveal ATP Interactions with the Unwound State of (CAG)_20_

**Figure 5:**
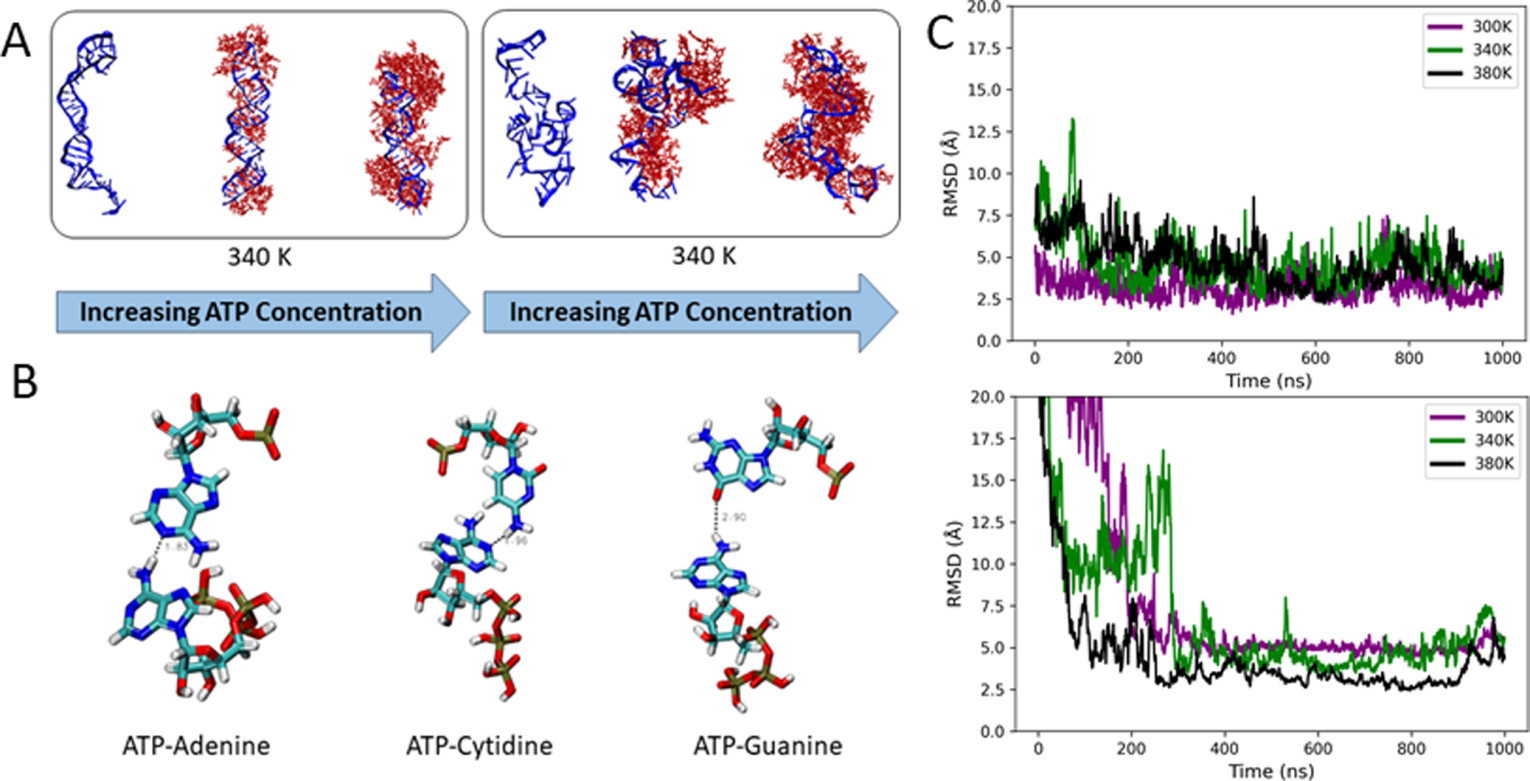
(A) Final structures from MD simulations of native (left) and unwound (CAG)_20_ RNA (right) at increasing ATP concentrations (left to right) from simulations at 340 K (see Figures S11 for other temperatures). ATP concentrations were 0, 5, and 10 mM. (B) Hydrogen bond formation between the ATP’s adenine residue and the RNA strand’s nucleobases adenine, cytidine, and guanine. (C) RMSD of native (top) and unwound states (bottom) of (CAG)_20_ RNA between each momentaneous configuration and the average structure over the course of the simulation with the 5 mM ATP concentration (see Figure S13 for other concentrations).

To investigate the mechanism of RNA destabilization by ATP, we performed large-scale molecular dynamics (MD) simulations. We used ATP and salt concentrations (see Table S4) and different temperatures (300, 340, and 380 K) to reproduce the experimental conditions. As even small RNAs of 50–80 nucleic acids tend to fold on the millisecond regime,^49^ it is currently impossible to simulate reversible (un)folding via conventional atomistic MD simulations, even though coarse-grained simulations are possible.^50^ Instead, we performed independent equilibrium MD simulations with two different starting conformations—the native, folded hairpin (named “native state” from here on) and a partially unfolded, unwound hairpin-like state (named “unwound state” hereon; see Figure S10). Final structures from all simulations with ATP molecules surrounding the RNA are shown in Figures 5A and S11. In the absence of ATP, native hairpin RNA was stable at 300 K but destabilized at 340 and 380 K. Specifically, with increasing temperature, there was greater variation in the chi torsion angles which characterizes the relative nucleobase/ribose orientation (Figure S12). In the unwound RNA, the presence of ATP led to fewer fluctuations in the root-mean-square-deviation (RMSD) with respect to the average nucleic backbone structure at 300 K (Figure S13). The time required for the RMSD to plateau is reduced in the presence of ATP at 300 and 380 K. This indicates that the backbone of the unwound RNA stabilized faster under these conditions. At 340 K and the 5 mM ATP concentration, the unwound RNA backbone also stabilized faster. However, with a 10 mM ATP concentration, the backbone RMSD stabilized after ∼450 ns, similar to that observed in the absence of ATP (Figure 5).

**Figure 6:**
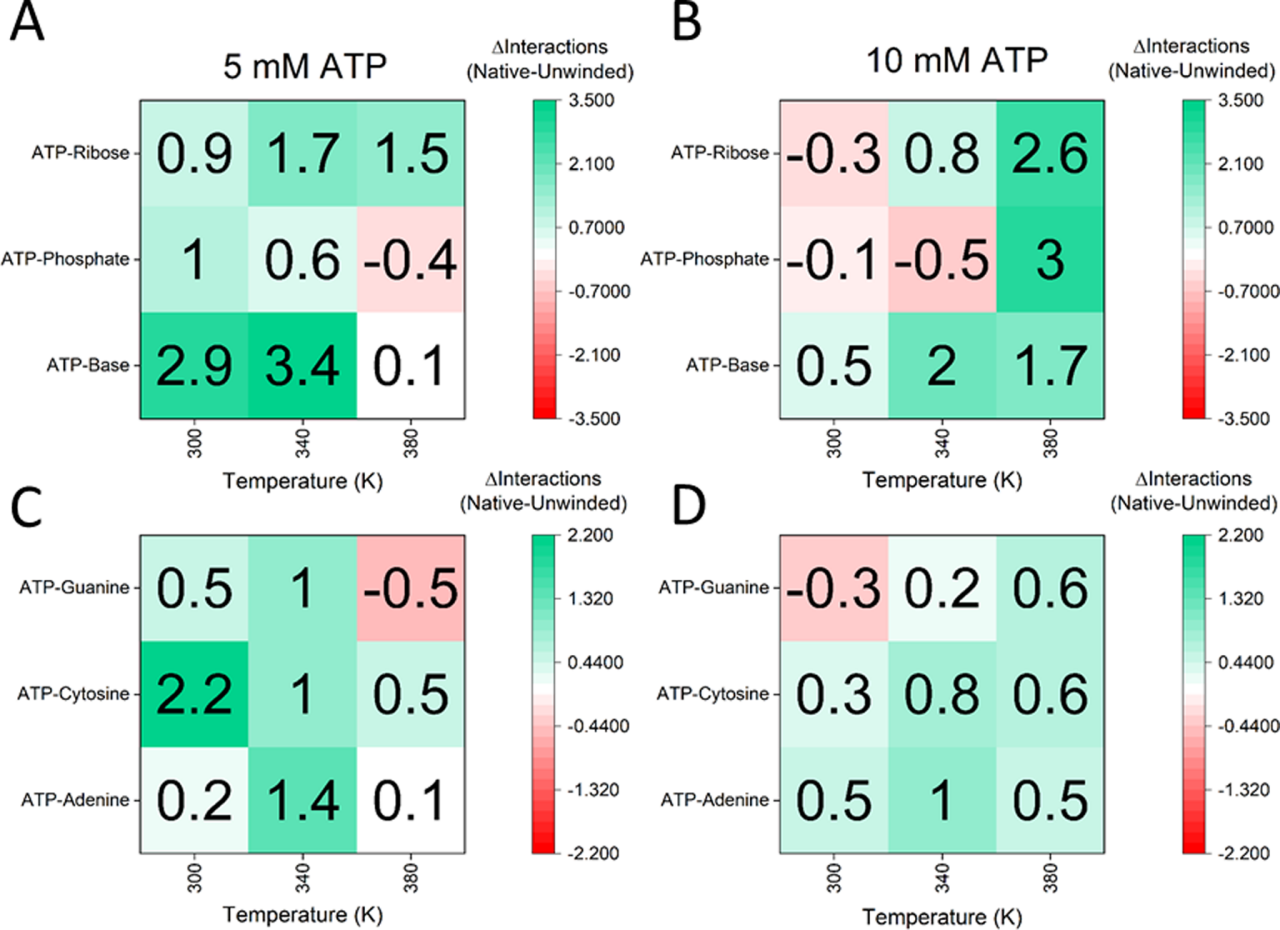
Changes in interaction of ATP with the (CAG)_20_ RNA’s ribose, phosphate, and base moieties between native and unwound states at 5 and 10 mM ATP and different temperatures. Average numbers of interactions between ATP and the different RNA moieties were counted, and differences between folded and unwound states were calculated [thresholds: distance <0.4 nm, angle (donor–hydrogen–acceptor) > 120°. Absolute values: see Table S5, Figure S14, and Figure S15]. (A,B) The main shift is observed in interactions between ATP and the nucleobases. Interactions with phosphate or ribose increase much less or even decrease. (C,D) ATP–base interactions from (A) and (B) broken down to the different nucleobases. ATP mainly interacts with adenine and cytosine.

We also observed an opening of the hairpin and exposition of the nucleobases for intermolecular binding interactions with ATP (see Figure 5B). Previous findings by Lambert and Draper also observed that urea destabilized RNA hairpin structures by preferential formation of H-bonds with the nucleobases, and similar results were reported for the interaction of ATP with proteins.^32,42^ This supports our notion that ATP replaces Watson–Crick base-pairing interactions, which subsequently leads to the enthalpic destabilization of the RNA backbone (note that increased temperature causes an entropic destabilization). To investigate and quantify this formation of hydrogen bonding interactions between ATP and RNA moieties, we used a donor–acceptor distance cutoff of 0.4 nm and a minimum donor–hydrogen–acceptor angle of 120° as the criteria (Figure 6, Table S5, and Figures S14 and S15). In all cases, ATP formed, on average, more hydrogen bonds with the atoms involved in Watson–Crick base pairing rather than with the RNA backbone’s phosphate or ribose moiety. Preferential formation of ATP–nucleobase hydrogen bonding interactions in the unwound state relative to the native state suggests that ATP destabilizes the RNA hairpin. An exception is at 5 mM ATP and 380 K, where the average number of ATP–nucleobase hydrogen bonds for native and unwound states was roughly the same. However, the average number of ATP–ribose hydrogen bonds in the unwound RNA was larger, and so the overall number of ATP-RNA interactions increased. The high local concentration of ATP close to the backbone also affects the surrounding hydration shell and biomolecular stability,^51−53^ as the phosphate groups of the backbone offer direct hydration sides and interactions sites for the counter ions and ATP.^52,53^ Quantifying these hydration shell changes would be exciting future work by complementing, e.g., small-angle X-ray scattering of time-averaged counterion distributions with MD simulations.^54^

To summarize, the MD simulations show an enthalpic destabilization of (CAG)_20_ RNA caused by preferential interactions of ATP with the nucleobases in the unwound state, which is different from an entropically stabilized unwound state caused by elevated temperatures. This finding is in line with results reported in the literature and further corroborates our experimental observations.

## Discussion

Sequestration and retention of RNA in nuclear speckles are a hallmark of several triplet repeat expansion diseases.^12^ Here, we investigated CAG-RNA recruitment to nuclear speckles under different cellular conditions as a function of RNA conformation and mobility. First, the comparison of (CAG)_20_ RNA with randomized and scrambled CAG sequences corroborated the CAG-repeat sequence specificity found in earlier studies.^15,55^ The comparison of (CAG)_20_ with HTT exon 1 RNAs showed that the flanking sequences slightly increased the colocalization with nuclear speckles and decreased the mobility. This could be explained by previous studies that suggested a profound role of the flanking sequences in HTT exon 1 folding, in particular suppressing CAG hairpin formation at low CAG repeat lengths.^9^ The authors suggested that a GCUGC moiety located in the 5′ UTR and the (CCG)_7-10_ and GCUGCUGC motifs in the flanking regions could provide additional binding valency to form an intermolecular RNA network. A high binding valency is commonly associated with biomolecules that engage with biomolecular condensates.^56^ Given the high RNA concentration inside the nuclear speckles,^12,57^ this would explain the decreased mobility and the increased association with nuclear speckles. Alternatively, the same binding valency could also be used to form extended hairpin structures which could then be bound by hairpin-binding proteins such as MBNL1 or MID1.^1,11^

An unexpected result was that both the recruitment and mobility of HTT exon 1 RNA were independent of CAG repeat length, with a similar behavior of constructs with repeat numbers below (*n* = 17) and above (*n* = 49, 72) the pathogenic threshold. This is, however, in agreement with fluorescence in situ hybridization studies reporting mostly small differences in the partitioning of CAG-RNA between the nucleoplasm and nuclear speckles across the pathogenic threshold.^10,15,55^ Significant differences were only observed for very high repeat expansions (>120) that are only rarely found in patients.^58,59^ Our results thus suggest that CAG repeat RNA recruitment to nuclear speckles itself may not be directly related to disease.^55^ However, pathogenic processes may still be induced by elongated repeats in speckles. Furthermore, the repeat threshold for such processes may be increased to higher repeat numbers due to somatic expansion and thus not detected in our experiments.^60^

Next, we investigated the conformations of CAG-repeat RNA recruited to nuclear speckles. It was expected that RNA may unfold in nuclear speckles due to its increased binding valency in the unfolded state. As such, previous studies found that single-stranded RNA^56^ and destabilized proteins^26^ are preferentially recruited by stress granules. Recruitment of the RNA in its unfolded state would lead to a significant decrease in folding stability in the nuclear speckles (e.g., Δ*G*_u_^θ,37^°^C^(nuclear speckles) < 0) in comparison to the nucleoplasm (can be shown in a thermodynamic cycle). However, at physiological temperature, we observed that folding stabilities were equal in both compartments with the RNAs being mostly folded (>90%), which suggests that no conformational transitions occur upon RNA migration. Heating led to further recruitment of the (CAG)_20_ RNA to nuclear speckles, increasing the PC, in particular near T_m_ with strongly increasing fractions of unfolded RNA. Importantly, we could not observe any homotypic interactions (entanglement, self-association) of unfolded (CAG)_20_ RNA. Thus, in contrast to the hypothesis by Jain and Vale,^15^ we think that aggregation and gelation of the CAG-RNA (although observed in vitro even with low CAG-repeat expansion) do not occur in cells and nuclear speckles. We suggest that CAG-RNA binding proteins like MBNL1^1^ or MID1^11^ could rather sequester the RNA in nuclear speckles. However, due to the high temperatures applied to the cells, this process could also be caused by non-physiological interactions such as the binding of aberrant temperature-unfolded proteins. Thus, experiments under different cell stresses are needed to further investigate this hypothesis.

The metabolite ATP emerged as a crucial factor to promote unfolding of RNAs [both for (CAG)_20_ and lm4U*] in the cell, with a CAG-specific function in enabling recruitment of the RNA to and its mobility in nuclear speckles. For the RNAs investigated here, MD simulations and in vitro experiments showed that the effect can be attributed to a direct interaction of the nucleobases with ATP molecules, rather than ATP acting as ‘a fuel’ for RNA-unwinding helicase activity.^15^ However, on the cellular level, it is important to note that the results do not exclude synergistic unfolding by helicases or unfolding by ATP-dependent RNA-binding proteins.

The function of ATP acting as a cosolute to maintain biomolecular processes in cells could explain why the cellular ATP concentration (5–10 mM)^46^ exceeds the level required to sustain energy-consuming processes by a factor of up to 1000.^25,42,43,61^ In line with this hypothesis, previous studies showed that elevated ATP levels could increase protein solubility and maintain the liquid-like properties of nucleoli,^62−64^ stress granules,^64,65^ and, in general, the cytoplasm of eukaryotes and prokaryotes.^42,61,64,66,67^

In this study, similar trends for folding stability in the presence of ATP were observed for both (CAG)_20_ and lm4U* RNA. However, quantitatively, the effects on (CAG)_20_ were 5-fold larger, despite the two constructs having a similar number of base pairs. This could be explained by the profound mismatches in the (CAG)_20_ hairpin which provide more space for ATP to interact with the RNA’s nucleobases. lm4U* does not contain such mismatches as it consists of longer stretches of dsRNA. Especially at low concentrations, ATP mainly interacts with the cytosine and adenine nucleobases, further corroborating this assumption. A remarkable fact is that in the case of lm4U*, the in-cell folding stability can be matched by cell-mimicking crowding agents, whereas for (CAG)_20_, ATP is additionally required. These findings suggest that ATP-induced destabilization occurs also in other hairpins. However, further studies are required to test this hypothesis and to investigate possible biological implications. Due to their comparable molecular composition,^42^ other nucleoside phosphates such as adenosine diphosphate, guanosine triphosphate, or cytidine 5′-triphosphate are expected to show similar effects, but their impact is assumed to be smaller due to their lower concentration in cells.

## Conclusions

The recruitment of RNA to nuclear speckles is specific to CAG-repeat RNA. Although the CAG hairpin is largely destabilized in cells, it remains folded in the cytoplasm, the nucleoplasm, and in nuclear speckles under physiological conditions. The metabolite ATP is crucial to destabilize the hairpin and maintain its mobility and nuclear speckle association by direct nucleobase interactions between ATP and RNA. Since mitochondrial function is affected in HD disease progression, leading to a decline in cellular ATP levels,^68,69^ the changes in CAG-RNA homeostasis could be linked to disease pathology. We hypothesize that the stabilized folded states of CAG-repeat RNA under ATP depletion could preferentially bind to transcription and translation factors such as MBNL1 and MID1, resulting in an upregulation of the expression of mutant HTT protein. In a self-amplifying mechanism, mutant HTT could enhance mitochondrial dysfunction, leading to a further decline in ATP levels. In fact, this mechanism would be most detrimental to striatal neurons (involved in HD disease pathology) with a high ATP content as these are most affected by decreasing ATP levels.^46,68,69^

## Supporting Information Available

Experimental procedures; sample sizes for post-hoc Tukey tests; fitting results; variation of the ATP concentration and position; summary of the average number of hydrogen bonds; sequences of RNA constructs; sequences of primers; number, size, and cumulative relative intensity of the foci; concentration dependency, results from single-molecule FRET experiments; secondary structures; acceptor fluorescence; local PCs and the PC ratio; background-corrected acceptor fluorescence intensity curves and D/A intensities; aggregation; relative change in the ATP concentration; final structures; torsion wheels; and RMSD (PDF)
