# Supplementary material for: CAG-Repeat RNA Hairpin Folding and Recruitment to Nuclear Speckles with a Pivotal Role of ATP as a Cosolute
Source: J Am Chem Soc. 2023 Apr 16;145(17):9571–83. doi: 10.1021/jacs.2c13653 (PMC10161218; doi:10.1021/jacs.2c13653)
Supplement: Supplementary file 1 — ja2c13653_si_001.pdf [file ja2c13653_si_001.pdf]

Supporting Information

CAG-Repeat RNA Hairpin Folding and Recruitment to Nuclear Speckles with a Pivotal Role of ATP as a Cosolute

Alexander Hautke,[1] Arthur Voronin,[2] Fathia Idiris,[2] Anton Riel,[1] Felix Lindner,[1] Amandine Lelièvre,[3] Jikang Zhu,[3] Bettina Appel,[3] Edoardo Fatti,[4] Karsten Weis,[4] Sabine Müller,[3] Alexander Schug,[5,6] Simon Ebbinghaus[1*]

[1] Institut für Physikalische und Theoretische Chemie, TU Braunschweig, Rebenring 56, 38106 Braunschweig, *corresponding author: s.ebbinghaus@tu-braunschweig.de

[2] Steinbuch Centre for Computing, Karlsruher Institut für Technologie, Herrmann-von-Helmholtz-Platz 1, 76344 Eggenstein-Leopoldshafen

[3] Institut für Biochemie, Universität Greifswald, Felix-Hausdorff-Straße 4, 17487 Greifswald

[4] Institut für Biochemie, ETH Zürich, Otto-Stern-Weg 3, 8093 Zürich, Schweiz

[5] Jülich Supercomputing Centre, Forschungszentrum Jülich, Wilhelm-Johnen-Str., 52452 Jülich

[6] Faculty of Biology, University of Duisburg/Essen, 45141 Essen

Materials and Methods

Chemicals

Rhodamine B, diethyl pyrocarbonate (DEPC), adenosine triphosphate (ATP), bovine serum albumin (BSA), acetic acid, methanol, Tween 20, glycine, sodium carbonate, copper sulfate (anhydrous), tris(3-hydroxypropyltriazolylmethyl)amine (THPTA), sodium phosphate and sodium ascorbate were all bought from Carl Roth. Dulbecco's modified eagle medium (DMEM), fetal bovine serum (FBS), penicillin-streptomycin mix, cOmplete™ mini protease inhibitor cocktail, potassium cyanide and 2-deoxyglucose were supplied by SigmaAldrich. From ThermoFisher Scientific, Dulbecco's phosphate-buffered saline (DPBS), M-PER™ mammalian protein extraction reagent, Opti-MEM, the Lipofectamine 3000 kit, ProLong Gold antifade medium with DAPI and the ULYSIS Alexa Fluor 594 nucleic acid labeling kit were bought. The PCR cleanup kit, Alexa Fluor 488 azide and Alexa Fluor 594 NHS ester were purchased from Jena Bioscience. RNasin plus RNase inhibitor and GoTaq master mix were supplied by Promega. The HiScribe T7

quick high yield RNA synthesis and Monarch RNA cleanup kits were both bought from New England Biolabs. Finally, Centri-Sep gel spin columns were supplied by Princeton Separations, SC35 nuclear speckles marker (ab11826) was purchased from Abcam, nuclease-free water and goat anti-mouse IgG antibodys labeled with Alexa Fluor 488 (A-11029) and Alexa Fluor 594 (A-11032) were bought from Invitrogen, ethanol for molecular biology was purchased form Merck Millipore and Leibovitz L-15 was bought from Gibco.

RNA Constructs

All RNA constructs used in this publication are listed in **Table S6** together with their respective manufacturers and sequences. Primer sequences used for PCR and *in vitro* transcription of HTT exon 1 RNA constructs are given in **Table S7**.

$(CAG)_{20}$ RNA labeled with Alexa Fluor 488 (donor, 5'-end), Alexa Fluor 594 (acceptor, 3'-end) or both fluorophores was synthesized, labeled, purified by gel electrophoresis and analyzed by MALDI by IBA GmbH, Göttingen. Scrambled $(CAG)_{20}$ RNA, Randomized 30% GC RNA and Randomized 50% GC RNA were synthesized by IBA GmbH, Göttingen. Afterwards, fluorescent labeling and purification by nuclease-free HPLC was performed using the following synthesis compositions: 6 nmol RNA, 100 nmol Alexa Fluor 488 azide, 300 nmol Alexa Fluor 594 NHS ester. All other dosages of chemicals and reaction steps were carried out according to the one-pot reaction protocol published by Gao et al.[1] After HPLC purification, the respective syntheses yielded separate fractions of the respective RNA construct labeled with Alexa Fluor 488 at the 5'-end, Alexa Fluor 594 at the 3'-end or both fluorophores in their respective positions. Only the Alexa Fluor 488-labeled fractions were used in the negative control experiments. Synthesis, purification and labelling of Alexa Fluor 488-AA$(CAG)_{10}(CUG)_{10}$AA-Alexa Fluor 594 RNA were carried out at Universität Greifswald following the protocols published previously.[1]

Plasmids containing HTT exon-1 sequences with 17, 49 and 72 CAG repeats inside an eGFP vector were a kind gift from the lab of Prof. Sybille Krauß (Universität Siegen). All HTT exon-1 RNA constructs were extracted from the plasmid via PCR (GoTaq master mix, Promega) and purified with a PCR cleanup kit (Jena Bioscience) according to the respective manufacturers' protocols. The integrity of the PCR product was confirmed by agarose gel electrophoresis, yielding one distinct band per construct (data not shown).

Afterwards, RNA was transcribed *in vitro* from the previously prepared DNA template using HiScribe T7 quick high yield RNA synthesis kit (New England Biolabs, NEB). The DNA template was digested with DNase 1 and purification of the RNA was conducted using Monarch RNA cleanup kit (NEB). All reactions were performed according to the manufacturers' protocols. Finally, the RNA constructs were precipitated with ethanol and statistically labeled with Alexa Fluor 594 using ULYSIS Alexa Fluor 594 nucleic acid labeling kit (ThermoFisher Scientific Inc.). The labeling reaction was carried out according to the manufacturer's protocol except for using only 1/5 of the originally intended amount of labelling reagent due to the large size of the RNA construct. The labeled product was purified on Centri-Sep gel spin columns (Princeton Separations Inc.) according to the manufacturer's protocol and concentrated by ethanol precipitation.

Preparation of RNA Solutions

$(CAG)_{20}$ RNA was received lyophilized and diluted in nuclease-free water (Invitrogen) to yield a 100 µM stock solution. The stock solution was directly used for microinjection. Scrambled $(CAG)_{20}$ RNA, Randomized 30% GC RNA and Randomized 50% GC RNA were dried on a SpeedVac after HPLC, precipitated from ethanol and diluted in nuclease-free water. Stock solutions had concentrations of 4800-6400 ng/µL and were directly used for microinjection. For HTT exon-1 constructs, stock solutions of varying concentrations (50-160 ng/µL) were directly used for microinjection as yielded from the final purification step.

To prepare $(CAG)_{20}$ RNA solutions for *in vitro* experiments, the stock solution was diluted in the cosolute solutions to a final concentration of 500 nmol/L. In case of DPBS-based (DPBS, pH 7.0-7.3: 2.7 mM KCl, 1.5 mM KH_2PO_4, 140 mM NaCl, 8 mM Na_2HPO_4) cosolute solutions, 1U/µL of RNasin plus RNase inhibitor (Promega) was added to the solution directly prior to measurement. All DPBS-based cosolute solutions were prepared from DEPC-treated DPBS.

In case of the experiments involving DEAD Box helicases, the $(CAG)_{20}$ stock solution was diluted in DEAD buffer solution (25 mM PIPES-KOH, pH 6.1-7.5; 130 mM KCl; 2 mM $MgCl_2$; 5 mM ATP/$MgCl_2$; 0.5 mM DTT). Final concentrations of $(CAG)_{20}$ and the different helicases were 500 nM, respectively.

Expression and Purification of DEAD Box Chaperones

Dhh1 (pKW5049), Ded1 (pKW5047), EIF4A (pKW2609), were expressed in E. coli Rosetta^{TM} (DE3) (Novagen) as 6xHis-TEV tagged proteins using an auto-induction medium. Cells were

grown at 37 °C in 1 L ZY complete medium to an OD_{600} of 0.7, then the temperature was lowered to 18 °C and growth continued for 20 h at 220 rpm. Cells were harvested, washed in cold PBS and the cell pellet was flash frozen in liquid nitrogen and stored at -20 °C until use. The cell pellet was dissolved in 10 ml/g of Lysis Buffer (20 mM HEPES-KOH pH 7.7, 500 mM KCl, 5 mM $MgCl_2$, 0.2 % NP-40, 10 mM Imidazole, 5 mM β-mercaptoethanol) supplemented with 0.5 mg/ml Lysozyme and 0.01 mg/ml DNase. and mechanically disrupted using a high pressure homogenizer (Emulsiflex C5, Avestin). The lysate was centrifuged at 20,000 rcf (SS-34 fixed angle rotor, Sorvall). The supernatant was filtered through a 0.45 µm PES membrane filter (Sarstedt) and incubated with Ni-NTA beads (Quiagen). Beads were washed with 5 column volumes of Detergent Buffer (20 mM HEPES-KOH pH 7.7, 500 mM KCl, 5 mM $MgCl_2$, 0.2 % NP-40, 10 mM Imidazole, 2 mM β-mercaptoethanol, 5 % Glycerol), High Salt Buffer (20 mM HEPES-KOH pH 7.7, 1.5 M KCl, 5 mM $MgCl_2$, 10 mM Imidazole, 2 mM β-mercaptoethanol, 5 % Glycerol), Imidazole Buffer (20 mM HEPES-KOH pH 7.7, 1.5 M KCl, 5 mM $MgCl_2$, 20 mM Imidazole, 2 mM β-mercaptoethanol, 5 % Glycerol), and eluted in 2.5 column volumes of Elution Buffer (20 mM HEPES-KOH pH 7.7, 500 mM KCl, 5 mM $MgCl_2$, 330 mM Imidazole, 2 mM β-mercaptoethanol, 5 % Glycerol). The elution buffer was exchanged to Imidazole Buffer by passing the proteins through a PD-10 column (GE Healthcare) and incubated overnight at 10 °C with 6xHis-TEV protease. The eluate was passed again through Ni-NTA beads to remove uncleaved proteins and the 6xHis-TEV protease, concentrated with centrifugal filter units (Millipore) and further purified by size exclusion chromatography on a Superdex 200 16/600 column (GE Healthcare) in SEC Buffer (30 mM HEPES-KOH pH 7.7, 500 mM KCl, 5 mM $MgCl_2$, 1 mM DTT, 10 % Glycerol) using an AKTA pure system (GE Healthcare). Positive fractions were pooled, further concentrated and final purity was assessed by SDS-PAGE and Coomassie stain (Instant Blue®, Abcam). 15 uL aliquots were flash frozen and stored at -80 °C.

$NOT1^{MIF4G}$ (pKW3469) was purified essentially as described above but with a modified Lysis Buffer (20 mM HEPES-KOH pH 7.7, 300 mM KCl, 5 mM $MgCl_2$, 2 mM β-mercaptoethanol) and SEC Buffer (30 mM HEPES-KOH pH 7.7, 300 mM KCl, 1 mM DTT, 10 % Glycerol). $Not1^{MIF4G}$ was purified by size exclusion chromatography on a Superdex 75 10/300 column (GE Healthcare).

Cell Culture and Preparation of HeLa Cell Lysate

HeLa cells were grown in T-25 culture flasks in DMEM supplemented with 10% FBS and 1% penicillin-streptavidin mix at 37 °C, 10% CO_2. Upon reaching 80-90% confluency, cells were

passaged every two (1:4) or every three (1:6) days. One or two days before measurement, cells were passaged into 35 mm round glass bottom dishes (FluoroDish, WPI).

To prepare HeLa cell lysate, cells were passaged into a 10 cm petri dish. The growth medium was removed and cells were washed once with DPBS. Afterwards, lysis was performed by adding 1000 µL of M-PER™ Mammalian Protein Extraction Reagent and 100 µL of cOmplete™, Mini Protease Inhibitor Cocktail. The dish was then shaken by hand for 5 min. The resulting suspension was transferred into an Eppendorf tube, shaken (10 min, 1,400 rpm), centrifuged (10 min, 14,000 g) and aliquoted for storage.

Determination of Relative Intracellular ATP Concentration

HeLa cells were cultured as described above. A sample culture was passaged to a six-well plate (Sarstedt) and transiently transfected with a DNA plasmid coding for the ATeam-nD/nA ATP sensor.[2] The sensor is a fusion protein consisting of the ε subunit of the bacterial F_0F_1-ATP-synthase centered between an N-terminal CFP and a C-terminal mVenus.

For transfection, 2 µg of plasmid DNA and 4 µL of P3000 reagent were diluted in 125 µL of Opti-MEM (ThermoFisher). In a second tube, 125 µL of Opti-MEM were supplemented with 4 µL of Lipofectamine 3000 reagent. Both solutions were incubated for 5 min and mixed. Afterwards, the resulting solution was supplemented to the HeLa cells' culturing medium and incubated for 6 h. Finally, cells were passaged to a FluoroDish and used for imaging the next day.

To determine the change of intracellular ATP conditions, the culturing medium was removed and cells were washed twice with DPBS. For the measurements, cells were kept in Leibovitz's L-15 supplemented with 30% FBS. Directly prior the measurement, stock solutions of 100 mM KCN and 1 M 2-deoxyglucose were added to the medium to achieve final concentrations of 1 mM KCN and 10 mM 2-deoxyglucose, respectively, and the medium was mixed thoroughly with a pipette. Cells were imaged on an Olympus FV3000 CLSM microscope with a 60x UPLFLN objective (Olympus). Separate images of donor and acceptor channels were recorded every 5 s for 60 min. Afterwards, the background was subtracted from every frame and the acceptor/donor (A/D) ratio was calculated to determine the relative change in ATP concentration.

Microinjection and Sample Preparation

Prior to microinjection, growth medium was removed, cells were washed twice with DPBS and medium was changed to Leibovitz's L-15 supplemented with 30% FBS. HeLa cells were

microinjected using an Eppendorf FemtoJet connected to an Eppendorf InjectMan NI2 micromanipulator. 2-3 µL of RNA stock solution in nuclease-free water were loaded into an Eppendorf FemtoTip II glass capillary using an Eppendorf Microloader pipette tip. Microinjection was carried out using the following settings: $p_i = 300 - 600$ hPa, $\Delta t_i = 0.1 - 0.2$ s, $p_h = 0 - 10$ hPa. Parameters were adjusted so that no visible expansion of cell volume did occur during microinjection. Cells were injected for 10-15 min and allowed to rest for 15 min afterwards. Afterwards, cells were sealed below a SecureSeal Imaging spacer (height = 0.12 mm, diameter = 13 mm, Grace Biolabs) and a coverslip (Marienfeld, diameter = 18 mm, #1) with 35 µL Leibovitz's L-15 supplemented with 30% FBS. For RNA depletion experiments, Leibovitz's L-15 supplemented with 30% FBS, 1 mM KCN and 10 mM 2-deoxyglucose was used. Cells were imaged within 30 min after microinjection. Cell viability was assessed by monitoring their morphology. Only healthy cells were used for FReI and FRAP experiments.

For temperature calibration and *in vitro* samples, a similar sample chamber was constructed. The changes applied are a spacer diameter of 9 mm and a sample volume of 14 µL.

Colocalization of CAG-repeat RNA with Nuclear Speckles

HeLa cells were cultured, transferred into glass bottom dishes, and microinjected with $(CAG)_{20}$ or one of the negative control RNAs labeled with Alexa Fluor 488 or HTT exon-1 RNA marked with Alexa Fluor 594 as described above. After microinjection, cells were allowed to rest for 15 min. All following incubation steps were performed in the dark in a humidity chamber. The injection medium was removed and cells were incubated for 10 min in methanol supplemented with 10% acetic acid for fixation and permeabilization. Afterwards, they were washed three times with DPBS for 5 min. To block unspecific staining, cells were incubated for 30 min in DPBS supplemented with 1% BSA, 22.52 g/L glycine and 0.1% Tween 20. The anti-SC35 nuclear speckles marker (Abcam, ab11826) was diluted in DPBS to a final concentration of 5 µg/mL and incubated on the cells (60 min, RT). Afterwards, cells were again washed three times with DPBS for 5 min to remove any unbound antibodies. A goat anti-mouse IgG antibody labeled with Alexa Fluor 488 (Invitrogen, A-11029, positive RNAs) or Alexa Fluor 594 (Invitrogen, A-11032, negative controls) was used as secondary antibody. It was diluted in DPBS to a final concentration of 10 µg/mL and incubated on the cells (60 min, RT). After a final washing step with DPBS (3x à 5 min), the medium was removed and cells were mounted between the bottom of the dish and a coverslip using ProLong Gold antifade mountant supplied with DAPI (Invitrogen). The mountant was allowed to cure over

night at RT. Confocal microscopy was performed on an Olympus FV3000 CLSM microscope using a 60x UPLSAPO S2 oil immersion objective one or two days after sample preparation. For longer term storage, samples were kept at -20°C. The three fluorophores were excited using lasers with a wavelength of 401 (DAPI/Nuclei), 488 (Alexa Fluor 488/Nuclear Speckles) and 561 nm (Alexa Fluor 594/RNA). Fluorophores were imaged separately and individual images for each channel were saved. Channel overlays were created afterwards using ImageJ FIJI.[3]

To quantitatively assess colocalization of the different RNA constructs with the nuclear speckles, an object-based approach was chosen. The analysis scheme employed a self-written CellProfiler[4–7] pipeline. Three channels were imported into the pipeline: Nuclei (DAPI), Nuclear Speckles (Alexa Fluor 488 for positive cases (HTT CAG 17/49/72 and $(CAG)_{20}$), Alexa Fluor 594 for negative controls (Scrambled $(CAG)_{20}$, Randomized 50% GC, Randomized 30% GC)) and RNA (Alexa Fluor 594 for positive cases, Alexa Fluor 488 for negative controls).

Channels were first aligned to one another to compensate for slight position differences between the three channels. Afterwards, illumination correction was performed to improve identification of nuclear speckles and RNA bodies in their respective channels. To achieve this, a median filter with an average object size of 60 pixels was generated for nuclear speckles and RNA channels and then subtracted from the raw data. In the next step, object identification was performed to identify nuclei, nuclear speckles and RNA bodies, respectively. To rule out any arbitrary spots that might have formed outside the nuclei, the "relate objects" function was used to only include nuclear speckles and RNA bodies localized within the boundaries of a nucleus. In the final step, the "relate objects" function was used to identify the RNA bodies overlapping with nuclear speckles and to calculate their fraction among the total number of RNA bodies.

Calculation of Local Partition Coefficients

The analysis scheme used here was derived from one published by Samanta et al.[8] All steps were carried out using a semiautomatic script in ImageJ FIJI.[3] Briefly, single frames corresponding to well-defined temperatures (37, 42, 55, 65, 37°C) along the FReI trajectory were extracted from the captured video files and ROIs were established for all nuclei. Nuclei of cells showing signs of apoptosis were manually excluded. After sharpening the image and subtracting the background, intensity values for all pixels outside the nuclei were set to 0.

Within the nuclei, ROIs for RNA foci were generated using a threshold and the ‘Analyze Particles’ function. Foci were automatically detected within preset ranges for size (1.0-10.0 μm^2) and circularity (0.1-1.0). ROIs for the focis’ immediate surroundings were generated using the ‘Enlarge’ and ‘XOR’ functions. Afterwards, average intensity values were calculated for both inside and outside the foci from an untreated image. The local partition coefficient (local PC) was calculated as the quotient of inside and outside fluorescence intensity.

Single Molecule FRET Experiments

Single molecule FRET (smFRET) experiments were performed using the Alba FFS system (ISS Inc.). Donor and acceptor fluorophores were excited using post-interleave excitation (PIE). To excite the donor (Alexa Fluor 488), a pulsed picosecond 488 nm laser (Chroma) was used. Excitation of the acceptor (Alexa Fluor 594) was achieved using a pulsed super-continuum laser (NKT Photonics SuperK Extreme) and a 594/10 band-pass filter. The excitation light was focused on the sample using an apochromatic water immersion objective (60x, NA = 1.2, Olympus). Fluorescence light was collected by the same objective. Donor and acceptor fluorescence were separated using a 560 nm long-pass filter and filtered using 525/40 (donor) and 647/94 (acceptor) band-pass filters (all Chroma). To block off-focus photons, 100 µm pinholes were placed in front of all detectors. Photons were detected by two avalanche photodiodes (Excelitas) and recorded using a time-correlated single-photon-counting module (ISS) in time-tagged time-resolved (TTTR) mode. Out of the single events, TCSPC histograms were calculated and afterwards analyzed in VistaVision (ISS) and OriginPro.

Fast Relaxation Imaging to Determine the Stability of $(CAG)_{20}$

The principles behind and the measurement technique of Fast Relaxation Imaging (FReI) have already been explained in previous publications.[9–11] In this study, measurements have been performed using a Zeiss AxioExplorer Z1 inverted fluorescence microscope equipped with a Colibri excitation system. Monochromatic Excitation light was guided towards a dichroic beam splitter (DFT 490+575, Zeiss) and then into a 40x objective (NA = 0.95, Zeiss). Fluorescence light from Donor and Acceptor channel was collected by the objective, guided through the first beam splitter and onwards to second beam splitter (BC 565, Zeiss) designed to separate donor and acceptor fluorescence. The two fluorescence channels were then individually imaged by one out of two CCD cameras (AxioCam HS, Zeiss). Each camera was equipped with an appropriate emission filter (BP 512/30 and BP 630/98, both Zeiss).

A continuous starting temperature was guaranteed by a peltier-heated temperature stage. Temperature jumps ($n = 12 - 16;\ \Delta T = 2.4 - 2.5\ °\mathrm{C}, \Delta t = 25$ s) were induced by an IR diode laser ($\lambda = 2{,}200$ nm, $P = 400$ mW, m2k Laser). After a starting period of 12 s during which no temperature jumps were administered, the IR laser was activated for the first temperature jump, homogeneously heating the cells and the complete field of view. Temperature jump amplitudes were calibrated using Rhodamine B. Fluorescence excitation intensity was kept constant during the whole measurement. IR laser intensity was controlled by a self-written LabView script. Images were recorded with 2 or 5 fps using AxioVision 4.8 (Zeiss) software.

Temperature calibration method exploited Rhodamine B's almost linear correlation of temperature and fluorescence quantum yield.[12] 14 µL of aqueous Rhodamine B solution (c = 100 µM) were sealed into a sample chamber as described in the *microinjection and sample preparation* section. The sample was then placed on the heating stage. Two measurements were performed. During the first measurement, 40 baseline frames were captured at 23 °C. The same sample was then heated up to 37 °C and measured as described in the *FReI – data acquisition* section. Temperature calibration measurements were performed once in the beginning of every measurement day. Data were saved as TIFF files and analyzed using ImageJ FIJI[3], a self-written Python script and Origin. In all cases, a rectangular region of interest was defined which encompassed most of the field of view, leaving out a narrow border at the edge of the frame. Average fluorescence intensity values were calculated for every frame. To calculate relative fluorescence intensity values, all values in the column were normalized to the video file's first value. To determine the exact starting temperature for measurements that started at 37 °C, they were also separately divided by the average baseline fluorescence intensity. Relative fluorescence intensities were converted into temperature values using **Equation 1** as published by Fu et al.[12] Here, $T(°C)$ is the temperature in °C and I is the normalized Rhodamine B fluorescence intensity.

$$T(°\mathrm{C}) = 149.15 - 317.84 \cdot I + 323.41 \cdot I^2 - 131.84 \cdot I^3$$

Equation 1: Equation used to convert relative Rhodamine B fluorescence intensity into temperature. T is the temperature in °C and I is the normalized fluorescence intensity.

For all *in vitro* and *in cell* FReI experiments, separate video files were saved for donor and acceptor channel. Data were saved as TIFF files and analyzed using ImageJ FIJI,[3] self-written Python scripts and Origin. Within every acquired video file, stationary regions of interest (ROIs) were defined for a background region with low fluorescence and each cell's cytosol and nucleus. In addition to that,

mobile ROIs for separately analyzing the RNA foci inside the nuclear speckles were defined using the TrackMate extension from the FIJI toolbox.[3] Donor and acceptor intensities were read out pixel-wise, averaged for each frame and ROI, and corrected by subtracting the background value. Afterwards, the donor-acceptor fluorescence intensity ratio D/A was calculated for each frame and ROI.

In measurements with no temperature jumps applied, it was shown that D/A decayed overtime, following the path of a single-exponential decay or a linear function (data not shown). This observation was attributed to photo-bleaching of the two fluorescent dyes. Thus, a single exponential decay or a linear function was fitted over the first 80 frames of each measurement and subtracted from the whole D/A trace for bleaching correction. The choice of the baseline function was made to fit the D/A trajectory in these 80 frames. Afterwards, each D/A trace was divided into respective time intervals of equal length using a self-written Python script. Each part equaled a single temperature jump's relaxation period ($\Delta t = 25$ s). The D/A relaxation curve after each temperature jump was fitted by single exponential relaxation kinetics using **Equation 2**, derived from the data analysis protocol published by Girdhar et al.[13]

$$B(t) = B(0) + A_u \cdot (1 - \exp(-kt)) - A_P \cdot t$$

Equation 2: Fitting equation for single temperature jumps. B(t) is the D/A signal at a given time point t, A_u is the unfolding-related signal amplitude, k is the rate constant of unfolding and A_P is the photo-bleaching signal amplitude within the single temperature jumps.

Here, $B(t)$ is the D/A signal at a given time point t, A_u is the unfolding-related signal amplitude, k is the rate constant of unfolding and A_a is the photo-bleaching amplitude. For some individual temperature jumps, fits did not fully converge by not reaching all the way down to the first data point while covering the rest of the curve well, resulting in too high values for $B(0)$ and too low values for A_u. In these cases, data were corrected by adding the difference between the first data point and the fit $B(0)$ value to A_u. Unfolding amplitudes A_u were extracted from the fit for each temperature jump, plotted to the corresponding temperature and fitted with **Equation 3** as published by Girdhar et al.[13] with $g^{(1)}$ being the first order cooperativity parameter, ΔT being the temperature jump amplitude, T_m being the melting temperature, R being the ideal gas constant and A_0 and m_A being the baseline intercept and slope, respectively. For all measurements, m_A was constrained to 0 and the change of heat capacity ΔC_p was assumed to be zero.

$$B(T) = -\frac{g^{(1)} \cdot \Delta T \cdot T_m}{R \cdot \left(T - \frac{\Delta T}{2}\right)^2} \cdot [A_0 + m_A \cdot (T - T_m)] \cdot \frac{\exp\left(-\frac{g^{(1)} \cdot \left(T - \frac{\Delta T}{2} - T_m\right)}{R \cdot \left(T - \frac{\Delta T}{2}\right)}\right)}{\left(1 + \exp\left(-\frac{g^{(1)} \cdot \left(T - \frac{\Delta T}{2} - T_m\right)}{R \cdot \left(T - \frac{\Delta T}{2}\right)}\right)\right)^2}$$

Equation 3: Better thermodynamics from kinetics equation. B(T) *is the resolved signal amplitude,* $g^{(1)}$ *is the first order cooperativity parameter,* ΔT *is the temperature jump amplitude,* T_m *is the melting temperature,* R *is the ideal gas constant,* A_0 *is the linear baseline offset and* m_A *is the linear baseline slope.*

The standard free energy of unfolding at 37 °C $\Delta G_u^{\theta,37°C}$ was calculated from $g^{(1)}$ and T_m according to **Equation 4**. Relations are given to convert $\Delta G_u^{\theta,37°C}$ into the equilibrium constant K and to calculate the folded and unfolded fractions at 37°C ($K^{37°C}, f_{folded}^{37\,°C}, f_{unfolded}^{37\,°C}$).

$$\Delta G_u^{\theta,37\,°C} = (310\ \mathrm{K} - T_m) \cdot g^{(1)}$$

$$K^{37°C} = \exp\left(-\frac{\Delta G_u^{\theta,37\,°C}}{R \cdot 310\ \mathrm{K}}\right)$$

$$f_{unfolded}^{37\,°C} = \frac{K^{37°C}}{1 + K^{37°C}}$$

$$f_{folded}^{37\,°C} = 1 - f_{unfolded}^{37\,°C}$$

Equation 4: Equation to calculate the standard free energy of unfolding $\Delta G_u^{\theta,T}$, equilibrium constant K and unfolded fraction $f_{unfolded}$ at a given temperature T out of melting temperature T_m and the first order cooperativity parameter $g^{(1)}$.

All errors are standard deviations calculated from the respective "Better biomolecule thermodynamics from kinetics" fits[13] or standard deviations that resulted from averaging. All errors were calculated using Origin. Gaussian error propagation was used if necessary.

FRAP Experiments for Studying RNA Mobility inside Foci

HeLa cells were microinjected RNA as described in the *microinjection and sample preparation* section. Measurements were performed on an Olympus FV3000 CLSM confocal microscope equipped with an OkoLab temperature chamber. The temperature chamber was heated to 42 °C to promote migration of the RNA into the nuclear speckles. Cells were incubated in the temperature chamber 10 min prior to measurement and imaged using an Olympus UPLSAPO 60x S2 oil

immersion objective and a laser with a wavelength of 561 nm at approx. 2 fps. ROIs were defined for a background region with low fluorescence outside of the cell, the nucleus and the bleached region. 25-30% of laser intensity were used for normal imaging of the cells while 100% were employed for bleaching the RNA foci. The bleaching process occurred in a time frame of 80-120 µs. Prior to the bleaching pulse, a baseline of approx. 5 s was recorded. After bleaching, the droplet fluorescence was allowed to recover for 30-50 s.

Video files were exported into TIFF files and analyzed using ImageJ FIJI[3] and Origin. Within the previously defined ROIs, fluorescence intensities were readout pixel-wise for every frame and averaged over the whole ROI. The data analysis was conducted according to Koulouras et al.'s protocol.[14] First, background correction was performed by subtracting the fluorescence intensity from the background ROI from that of nucleus and bleached region. Afterwards, the bleaching curve was normalized using **Equation 5** and **Equation 6**. Here, $I_{dnorm,bl}(t)$ is the double-normalized fluorescence intensity of the bleached region corrected for initial intensity differences in the bleached region and differences in total fluorescence over the course of the measurement. n_{pre} is the number of frames acquired before the bleaching pulse, $I_{nuc}(t)$ is the non-normalized fluorescence intensity of the nucleus ROI and $I_{bl}(t)$ is the non-normalized fluorescence intensity of the bleached region ROI. $I_{fnorm,bl}(t)$ is the full-scale normalized fluorescence intensity of the bleached region. Compared to $I_{dnorm,bl}(t)$, it is additionally corrected for differences in bleaching depth. t_{post} is the time point of the first frame after the bleaching pulse.

$$I_{dnorm,bl}(t) = \left(\frac{\frac{1}{n_{pre}} \cdot \sum_{t=1}^{n_{pre}} I_{nuc}(t)}{I_{nuc}(t)} \right) \cdot \left(\frac{I_{bl}(t)}{\frac{1}{n_{pre}} \cdot \sum_{t=1}^{n_{pre}} I_{bl}(t)} \right)$$

Equation 5: Equation used for double normalization of the intensity values as published by Koulouras et al.[14] Here, $I_{dnorm}(t)$ is the double-normalized fluorescence intensity corrected for initial intensity differences in the bleached region and differences in total fluorescence over the course of the measurement. n_{pre} is the number of frames acquired before the bleaching pulse, $I_{nuc}(t)$ is the fluorescence intensity acquired in the nucleus ROI and $I_{bl}(t)$ is the fluorescence intensity acquired in the bleached region ROI.

$$I_{fnorm,bl}(t) = \frac{I_{dnorm,bl}(t) - I_{dnorm,bl}(t_{post})}{1 - I_{dnorm,bl}(t_{post})}$$

Equation 6: Equation used for full-scale normalization of the intensity values as published by Koulouras et al.[14] $I_{fnorm,bl}(t)$ is the full-scale normalized fluorescence intensity of the bleached region. It is additionally corrected for differences in bleaching depth. t_{post} is the time point of the first frame after the bleaching pulse.

Finally, the full-scale normalized FRAP curves were fitted with a single exponential function according to **Equation 7** and mobile and immobile fractions were calculated using **Equation 8**. Here, I_0 is the initial intensity of the first data point after the bleaching pulse, A is the recovery amplitude and k is the recovery rate constant. f_{mobile} and $f_{immobile}$ are the calculated mobile and immobile fractions, respectively.

$$I_{fnorm,bl}(t) = I_0 + A \cdot (1 - \exp(-kt))$$

Equation 7: Equation used for fitting the full-scale normalized FRAP curves with I_0 being the initial intensity of the first data point after the bleaching pulse, A being the recovery amplitude and k being the recovery rate constant.

$$f_{mobile} = \frac{A}{1 - I_0}; \; f_{immobile} = 1 - f_{mobile}$$

Equation 8: Equation used for calculating mobile fraction f_{mobile} and immobile fraction $f_{immobile}$.

MD Simulations for Gaining Mechanistical Insight into CAG-repeat RNA's Destabilization by ATP

All MD simulations were performed using GROMACS 2020.[15,16] We used the explicit water model tip3p[17] and the feb2021 version of the CHARMM36[18,19] force field for RNA with CHARMM General Force Field[20] (CGENFF) parameterization for ATP. ATP concentrations (see **Table S3**) and ion concentrations including counter ions (3 mM KCl, 140 mM NaCl) were set in equivalent to the concentrations in our wet-lab experiments. We used cube-shaped systems boxes with a side length of a = 24.7 nm with periodic boundary conditions (PBC). The box contained about $1.53 \cdot 10^6$ atoms. Such a large box size provided sufficient space to accommodate large scale RNA motions without self-interactions from PBC. For equilibration, NVT and NPT simulations were performed for 100 ps each at the respective temperatures prior to the MD production simulations. For all simulations, we used a leap-frog integrator, V-rescale thermostat and Parrinello-Rahman barostat. Additional details for the simulation setup can be found in the attached GROMACS.mdp files.

We selected two different RNA conformations (see **Figure S5**) as starting points for the simulations. The first starting conformation represented the native hairpin state ("native state") and

was modeled with RNAComposer.[21,22] The second starting conformation resembled an unwinded version of the native molecule (“unwinded state”) and was modeled with pyRosetta.[23] This conformation had no initial bonds between the base pairs while maintaining a compact spatial shape. In total, we performed 18 simulations with trajectory times of 1 µs each, by varying the starting conformation of $(CAG)_{20}$ RNA (native, unwinded), the ATP concentration (no, medium, high) and the system temperature (300 K, 340 K, 380 K).

Supplementary Tables

Table S1: Sample sizes for post-hoc Tukey tests as shown in Figure 1B), 1D), 1E), 1F), 2C), 3A) and 3B) and others.

Construct	Sample Size Colocalization (Cells)	Sample Size FRAP (Foci)	Sample Size FReI (Cells/Foci)	Sample Size PC (Cells)
HTT CAG 17	104	73	-	-
HTT CAG 17, ATP-depleted	-	55	-	-
HTT CAG 49	45	86	-	-
HTT CAG 49, ATP-depleted	-	61	-	-
HTT CAG 72	108	58	-	-
HTT CAG 72, ATP-depleted	-	51	-	-
$(CAG)_{20}$ 100 µM stock solution	147	114	-	-
$(CAG)_{20}$ 10 µM stock solution	-	111	-	-
$(CAG)_{20}$ 300 µM stock solution	-	103	-	-
$(CAG)_{20}$ 100 µM stock solution, ATP-depleted	-	166	-	-
Scrambled $(CAG)_{20}$	164	117	-	-
Randomized 50% GC	111	101	-	-

Randomized 30% GC	28	108	-	-
$(CAG)_{20}$ Cytosol	-	-	40	-
$(CAG)_{20}$ Cytosol, ATP-depleted	-	-	12	-
$(CAG)_{20}$ Nuclei	-	-	16	-
$(CAG)_{20}$ Nuclei, ATP-depleted	-	-	14	-
$(CAG)_{20}$ Nuclear Speckles	-	-	35	-
$(CAG)_{20}$ 37°C (T_0)	-	-	-	30
$(CAG)_{20}$ 42°C	-	-	-	30
$(CAG)_{20}$ 55°C	-	-	-	30
$(CAG)_{20}$ 65°C	-	-	-	21
$(CAG)_{20}$ 37°C (T_∞)	-	-	-	30
$(CAG)_{20}$ 37°C (T_0), ATP-depleted	-	-	-	20
$(CAG)_{20}$ 42°C, ATP-depleted	-	-	-	19
$(CAG)_{20}$ 55°C, ATP-depleted	-	-	-	20
$(CAG)_{20}$ 65°C, ATP-depleted	-	-	-	19
$(CAG)_{20}$ 37°C (T_∞), ATP-depleted	-	-	-	19

Table S2: Fitting results (mean ± s.d.) for FReI experiments surveying $(CAG)_{20}$ *in vitro* and in HeLa cells. Sample sizes are provided in parentheses in the "Region/Solution" column. Note that DEAD buffer contains 5 mM ATP.

Region/Solution (sample size n)	T_m(K)	$g^{(1)}$ (J/(K · mol))	$\Delta G_u^{\theta,37\,°C}$ (kJ/mol)	K	$f_{unfolded}$(%)

DPBS (4)	348.8 ± 1.7	-540 ± 50	20.7 ± 1.0	0.0003	0.03
DPBS + 5 mM ATP (4)	349.7 ± 4.5	-500 ± 80	19.6 ± 0.5	0.0005	0.05
DPBS + 7.5 mM ATP (5)	363.1 ± 8.7	-320 ± 60	16.9 ± 1.2	0,0014	0.14
DPBS + 10 mM ATP (4)	337.4 ± 0.8	-460 ± 10	12.5 ± 0.3	0,0079	0.78
DPBS + 10 mM ATP + 300 g/L PEG 200 (4)	336.9 ± 2.1	-210 ± 10	5.6 ± 0.2	0.1142	10.25
DPBS + 10 mM ATP + 300 g/L PEG 2000 (4)	341.4 ± 1.2	-230 ± 20	7.3 ± 0.3	0.0591	5.58
DPBS + 10 mM ATP + 300 g/L PEG 8000 (4)	336.4 ± 0.4	-180 ± 10	3.9 ± 1.4	0.2206	18.07
DPBS + 10 mM ATP + 300 g/L Sucrose (4)	327.9 ± 0.4	-250 ± 10	4.5 ± 0.2	0.1749	14.88
HeLa cell lysate (4)	351.3 ± 3.8	-370 ± 60	15.1 ± 0.8	0.0029	0.29
DEAD (5)	360.6 ± 5.6	-340 ± 70	17.0 ± 2.0	0.0014	0.14
DEAD + 5 mM ATP (9)	366.4 ± 8.8	-300 ± 40	16.5 ± 1.2	0.0017	0.17
DEAD + 5 mM ATP + 500 nM Ded1 (7)	364.7 ± 8.2	-370 ± 70	19.7 ± 1.4	0.0004	0.05
DEAD + 5 mM ATP + 500 nM Dhh1/NOT1 (7)	361.6 ± 5.5	-340 ± 50	17.3 ± 1.1	0.0012	0.12

DEAD + 5 mM ATP + 500 nM EIF4A (7)	358.7 ± 2.9	-360 ± 40	17.3 ± 1.5	0.0012	0.12
HeLa cell cytosol (16)	330.7 ± 5.3	-370 ± 100	6.8 ± 2.1	0.0717	6.69
HeLa cell cytosol, ATP-depleted (12)	353 ± 11	-210 ± 50	8.5 ± 0.8	0.0371	3.58
HeLa cell nucleus (39)	326.5 ± 3.8	-400 ± 120	6.0 ± 1.4	0.0978	8.91
HeLa cell nucleus, ATP-depleted (14)	350 ± 12	-190 ± 30	7.5 ± 1.5	0.0547	5.18
HeLa cell nuclear speckles (35)	325.4 ± 3.7	-420 ± 110	6.4 ± 1.6	0.0837	7.73

Table S3: Fitting results (mean ± s.d.) for FReI experiments surveying lm4U* *in vitro* and in HeLa cells. Sample sizes are provided in parentheses in the "Region/Solution" column. Note that DEAD buffer contains 5 mM ATP.

Region/Solution (sample size n)	T_m(K)	$g^{(1)}$ (J/(K · mol))	$\Delta G_u^{\theta,37\,°C}$ (kJ/mol)	K	$f_{unfolded}$(%)
DPBS (10)	310.5 ± 0.9	-640 ± 40	0.3 ± 0.6	0.8901	47.09
DPBS + 5 mM ATP (4)	312.6 ± 2.5	-560 ± 90	1.3 ± 1.0	0.6039	37.65
DPBS + 10 mM ATP (4)	308.2 ± 1.2	-650 ± 40	-1.2 ± 0.9	1.5929	61.43
DPBS + 5 mM Adenosin (4)	312.6 ± 2.5	-560 ± 90	3.0 ± 0.3	0.3123	28.27
DPBS + 10 mM Adenosin (4)	308.2 ± 1.2	-650 ± 40	2.4 ± 0.3	0.3941	23.80
DEAD (4)	324.8 ± 0.9	-430 ± 30	6.3 ± 0.8	0.0858	7.99

DEAD + 500 nM Ded1 (4)	323.7 ± 2.5	-490 ± 70	6.6 ± 0.5	0.0773	7.17
DEAD + 500 nM Dhh1/NOT1 (4)	325.3 ± 4.0	-490 ± 80	7.2 ± 0.7	0.0612	5.77
DEAD + 500 nM EIF4A (4)	324.3 ± 2.3	-510 ± 80	7.2 ± 0.6	0.0612	5.77
HeLa cell cytosol (26)	305.8 ± 4.0	-320 ± 80	-1.3 ± 1.0	1.6559	62.35
HeLa cell cytosol, ATP-depleted (27)	306.1 ± 5.0	-260 ± 90	-0.8 ± 1.1	1.3639	57.70
HeLa cell nucleus, (25)	301.1 ± 4.4	-260 ± 70	-2.1 ± 0.8	2.2586	69.31
HeLa cell nucleus, ATP-depleted (26)	305.2 ± 3.9	-250 ± 50	-1.0 ± 1.1	1.4740	59.58

Table S4: Variation of ATP concentration and positioning. The number of ATP molecules were scaled to system size. ATP is positioned at the start of simulation either centered (around RNA molecule) or random within the remaining system box.

**Case**	**ATP concentration (mM)**	**Total number of ATPs**	**Number of centered ATPs**	**Number of random ATPs**
No	0	0	0	0
Medium	5	50	20	30
High	10	100	40	60

Table S5: Summary of the average number of hydrogen bonds formed between ATP and RNA moieties. Interactions with the nucleobases are also separated into the different types of bases.

**RNA**	**ATP Conc.**	**Temperature (K)**	**Average # of ATP-Base**	**Average # of ATP-ADE**	**Average # of ATP-CYT**	**Average # of ATP-GUA**	**Average # of ATP-Phosph.**	**Average # of ATP-Ribose**
Native	10 mM	300	4.5	1.5	1.0	2.0	4.3	1.2
Native	10 mM	340	3.1	1.9	0.6	0.6	3.0	1.5
Native	10 mM	380	5.0	1.8	1.6	1.6	2.9	2.4
Native	5 mM	300	2.6	1.8	0.2	0.6	1.1	0.6
Native	5 mM	340	2.9	0.9	1.6	0.4	1.5	0.4
Native	5 mM	380	4.4	1.5	0.9	2.0	3.8	1.5
Unwound	10 mM	300	5.0 (↑ 0.5)	2.0 (↑ 0.5)	1.3 (↑ 0.3)	1.7 (↓ 0.3)	4.2 (↓ 0.1)	0.9 (↓ 0.3)
Unwound	10 mM	340	5.1 (↑ 2.0)	2.9 (↑ 1.0)	1.4 (↑ 0.8)	0.8 (↑ 0.2)	2.5 (↓ 0.5)	2.3 (↑ 0.8)
Unwound	10 mM	380	6.7 (↑ 1.7)	2.3 (↑ 0.5)	2.2 (↑ 0.6)	2.2 (↑ 0.6)	5.9 (↑ 3.0)	5.0 (↑ 2.6)
Unwound	5 mM	300	5.5 (↑ 2.9)	2.0 (↑ 0.2)	2.4 (↑ 2.2)	1.1 (↑ 0.5)	2.1 (↑ 1.0)	1.5 (↑ 0.9)
Unwound	5 mM	340	6.3 (↑ 3.4)	2.3 (↑ 1.4)	2.6 (↑ 1.0)	1.4 (↑ 1.0)	2.1 (↑ 0.6)	2.1 (↑ 1.7)
Unwound	5 mM	380	4.5 (↑ 0.1)	1.6 (↑ 0.1)	1.4 (↑ 0.5)	1.5 (↓ 0.5)	3.4 (↓ 0.4)	3.0 (↑ 1.5)

Table S6: Sequences of RNA constructs.

Abbreviation	Synthesis	Sequence
mono-labeled $(CAG)_{20}$	IBA GmbH	AACAGCAGCAGCAGCAGCAGCAGCAGCAGCAGC AGCAGCAGCAGCAGCAGCAGCAGCAGCAGAA-C6 Linker-Alexa Fluor 594
dual-labeled $(CAG)_{20}$	IBA GmbH	Alexa Fluor 488-C6 Linker-AACAGCAGCAGCAGCAG CAGCAGCAGCAGCAGCAGCAGCAGCAGCAGCAG CAGCAGCAGCAGAA-C6 Linker-Alexa Fluor 594
lm4U*	IBA GmbH	Alexa Fluor 488-C6 Linker-GUUGAACUUUUGAAUA GUGAUUUAGGAGGUUAAU-C6 Linker-Alexa Fluor 594

Scrambled $(CAG)_{20}$	IBA GmbH; Müller Lab, Greifswald University	Alexa Fluor 488-C6 Linker-AAGCCCAAGGCACCGAA CGCCCAGCACGGGACGGAAGGAGAGGCAACAG ACCGGACAGACCAAA
Randomized 30% GC	IBA GmbH; Müller Lab, Greifswald University	Alexa Fluor 488-C6 Linker-AAUUAUAUGCACAGUG UGCUAACUAUAUAUAGAUAGAAAUCAUAGGCG AGUUUAAUUUUUGAAA
Randomized 50% GC	IBA GmbH; Müller Lab, Greifswald University	Alexa Fluor 488-C6 Linker-AAUCUUAUUAAGCUUA UAGAAAAAUCCAUACCAGCGAUUUCCAGAUUU CUUCGCACUAACGGAA
HTT CAG 17	PCR and *in vitro* transcription. Plasmids: Sybille Krauss, DZNE	GACCCTGGAAAAGCTGATGAAGGCCTTCGAGTC CCTCAAGTCCTTCCAGCAGCAGCAGCAGCAGCA GCAGCAGCAGCAGCAGCAGCAGCAGCAGCAGCA GCAGCAGCAGCAGCAGCAGCAGCAGCAGCAGCA GCAGCAGCAGCAGCAGCAGCAGCAGCAGCAGCA GCAGCAGCAGCAGCAGCAGCAGCAGCAGCAGCA GCAGCAGCAGCAGCAGCAGCAGCAGCAGCAGCA GCAGCAGCAGCAGCAGCAGCAGCAGCAACAGCC GCCACCGCCGCCGCCGCCGCCGCCGCCTCCTCAG CTTCCTCAGCCGCCGCCGCAGGCACAGCCGCTGC TGCCTCAGCCGCAGCCGCCCCCGCCGCCGCCCCC GCCGCCACCCGGCCCGGCTGTGGCTGAGGAGCC GCTGCACCGACC
HTT CAG 49	PCR and *in vitro* transcription. Plasmids: Sybille Krauss, DZNE	GACCCTGGAAAAGCTGATGAAGGCCTTCGAGTC CCTCAAGTCCTTCCAGCAGCAGCAGCAGCAGCA GCAGCAGCAGCAGCAGCAGCAGCAGCAGCAGCA GCAGCAGCAGCAGCAGCAGCAGCAGCAGCAGCA GCAGCAGCAGCAGCAGCAGCAGCAGCAGCAGCA GCAGCAGCAGCAGCAGCAGCAGCAACAGCCGCC

		ACCGCCGCCGCCGCCGCCGCCGCCTCCTCAGCTT CCTCAGCCGCCGCCGCAGGCACAGCCGCTGCTGC CTCAGCCGCAGCCGCCCCCGCCGCCGCCCCCGCC GCCACCCGGCCCGGCTGTGGCTGAGGAGCCGCT GCACCGACC
HTT CAG 72	Self-made by PCR and *in vitro* transcription. Plasmids: Sybille Krauss, DZNE	GACCCTGGAAAAGCTGATGAAGGCCTTCGAGTC CCTCAAGTCCTTCCAGCAGCAGCAGCAGCAGCA GCAGCAGCAGCAGCAGCAGCAGCAGCAGCAGCA GCAGCAGCAGCAGCAGCAGCAGCAGCAGCAGCA GCAGCAGCAGCAGCAGCAGCAGCAGCAGCAGCA GCAGCAGCAGCAGCAGCAGCAGCAGCAGCAGCA GCAGCAGCAGCAGCAGCAGCAGCAGCAGCAGCA GCAGCAGCAGCAGCAGCAGCAGCAGCAACAGCC GCCACCGCCGCCGCCGCCGCCGCCGCCTCCTCAG CTTCCTCAGCCGCCGCCGCAGGCACAGCCGCTGC TGCCTCAGCCGCAGCCGCCCCCGCCGCCGCCCCC GCCGCCACCCGGCCCGGCTGTGGCTGAGGAGCC GCTGCACCGACC

Table S7: Sequences of primers used for PCR and *in vitro* transcription of HTT CAG 17/49/72.

Primer	Sequence
HTT T7 forward primer	CCAAGCTTCTAATACGACTCACTATA GGGAGAATGGCGGACCCTGGAAAAGCTCATGAAGG
HTT reverse primer	GGTCGGTGCAGCGGCTCCTCAGC

Supplementary Figures

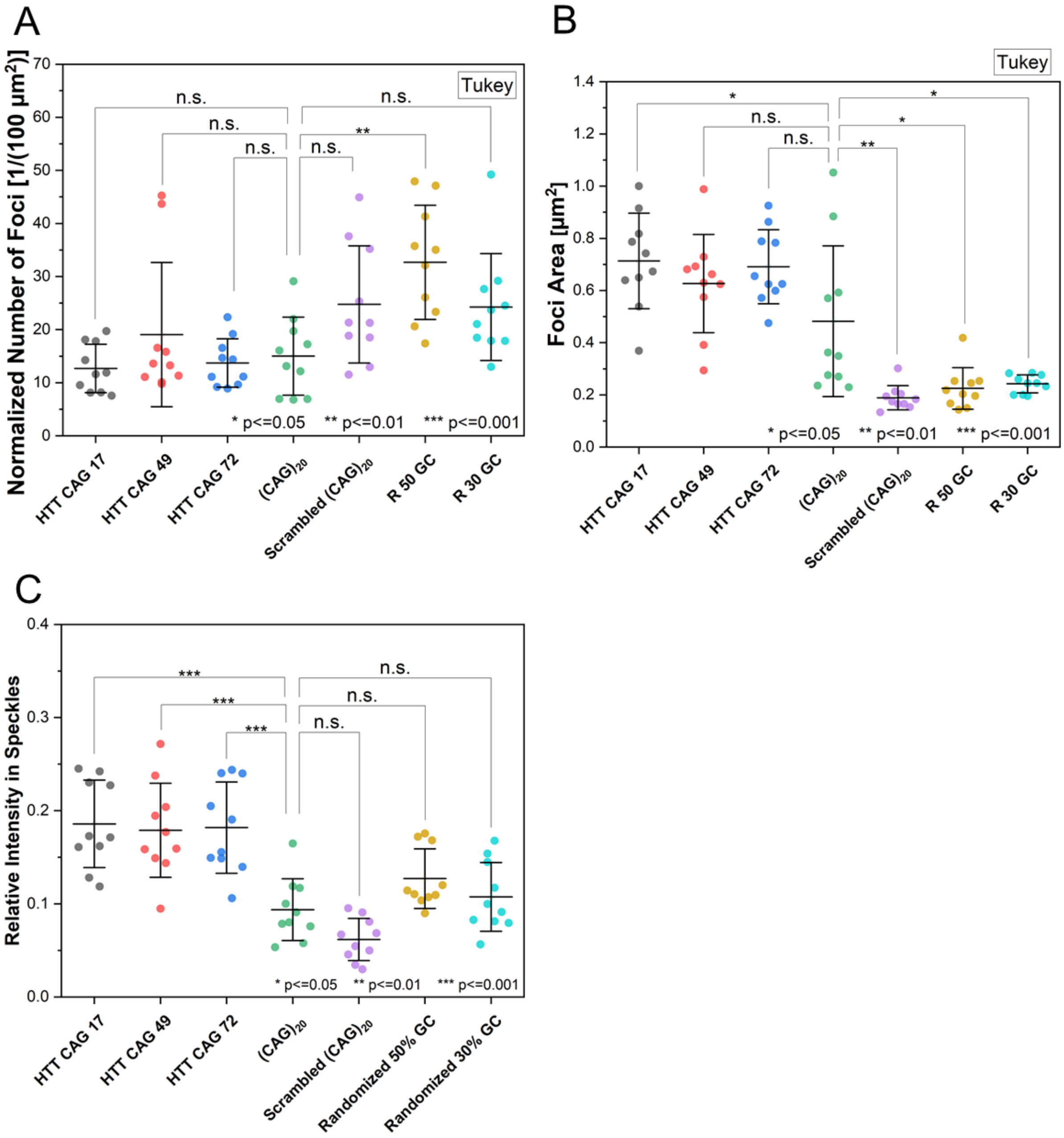

Figure S1: Number of RNA foci normalized to nuclear area (A), size of RNA foci (B) and cumulated relative intensity of the foci compared to the whole nucleus (C) for different RNAs. Statistical analysis was performed by a one-way ANOVA and post-hoc Tukey test (n = 10). Error bars show standard deviation calculated by Gaussian error propagation (see *materials and methods* section).

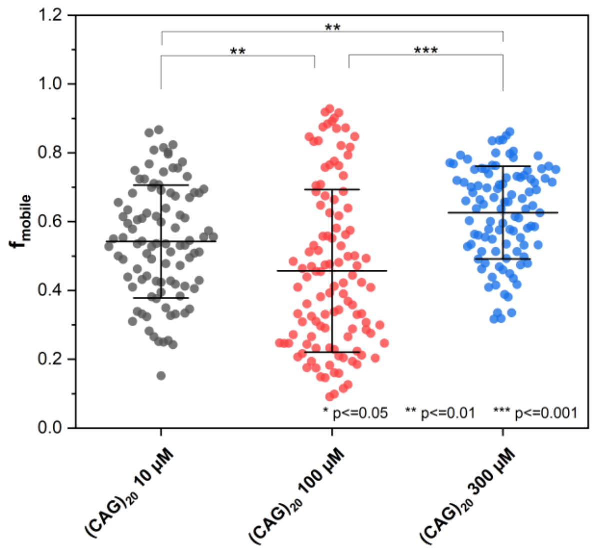

Figure S2: Concentration dependency of mobile fraction in FRAP experiments. HeLa cells were microinjected with differently concentrated $(CAG)_{20}$ stock solutions and FRAP experiments were performed. Results are as follows: $c_0 = 10\ \mu M: 54 \pm 16\%$; $c_0 = 100\ \mu M: 46 \pm 24\%$; $c_0 = 300\ \mu M: 62 \pm 13\%$.

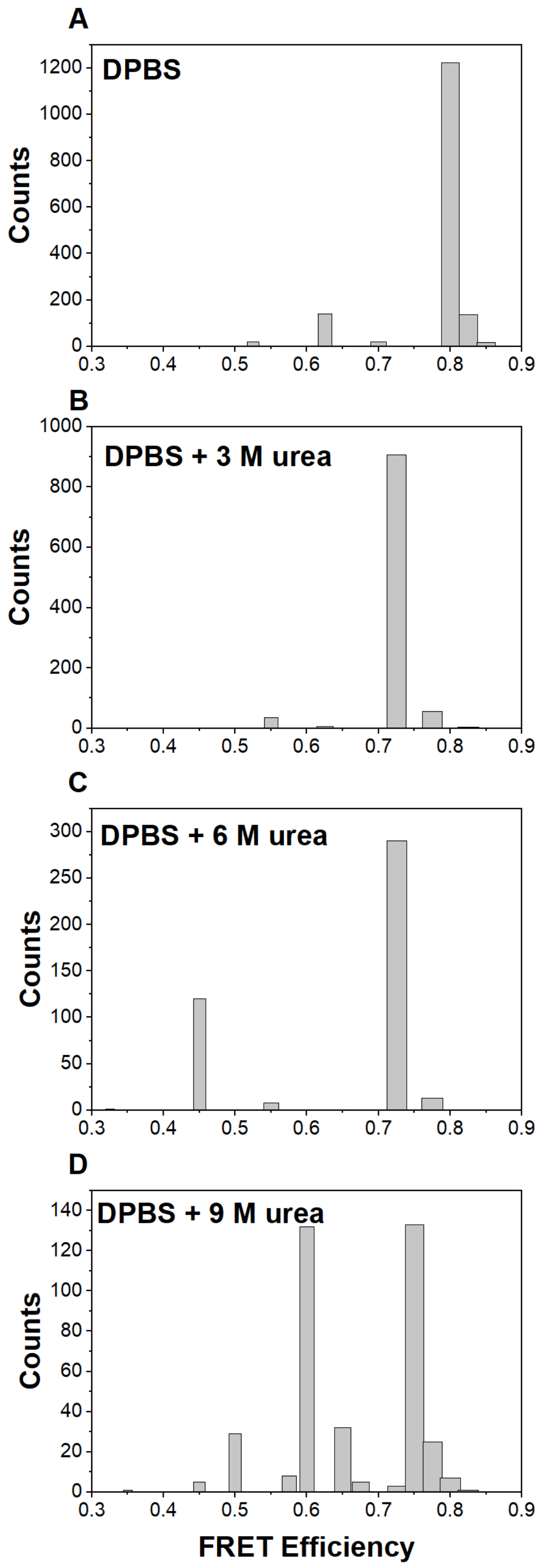
A
DPBS
B
DPBS + 3 M urea
C
DPBS + 6 M urea
D
DPBS + 9 M urea
Counts
FRET Efficiency

Figure S3: Results from single molecule FRET experiments with $(CAG)_{20}$ under native (DPBS, A) and increasingly denaturing (DPBS + 3 M / 6 M / 9 M urea) conditions. All experiments show a narrow peak at high FRET (0.7-0.8) resembling the native state and a much broader peak at lowered FRET (0.5-0.6) resembling the unfolded state.

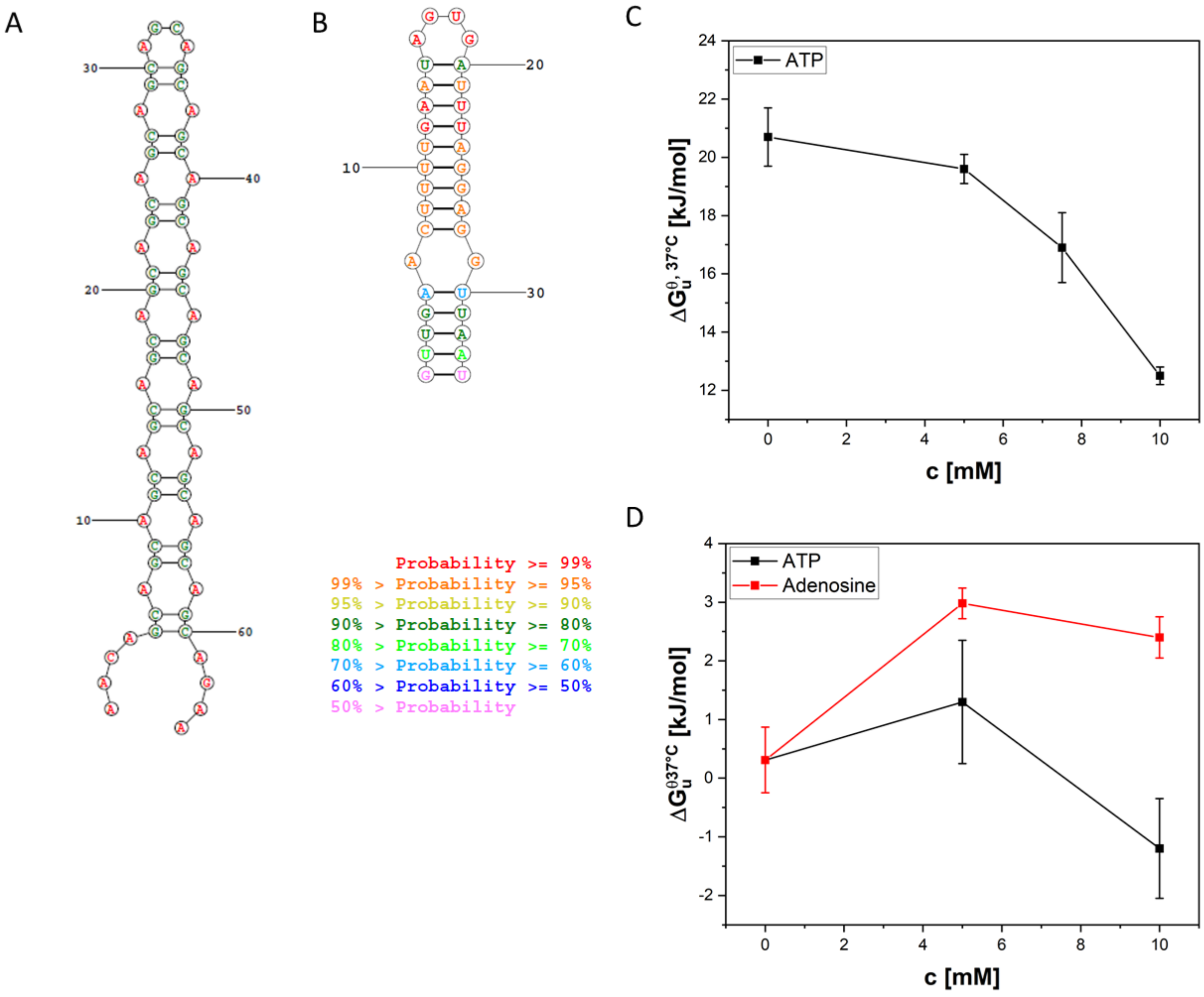

Figure S4: A, B) Secondary structures of $(CAG)_{20}$ (A) and lm4U* (B) as calculated by the "RNAstructure" server.[24] Colors resemble probabilities as shown below panel B. C, D) $\Delta G_u^{\theta,37°C}$ of $(CAG)_{20}$ (A) and lm4U* (B) plotted against concentration of ATP (black) and adenosine (red). A non-linear behavior was observed in both cases. Measurements with Adenosine were also attempted for $(CAG)_{20}$ but melting points were too high to be resolved.

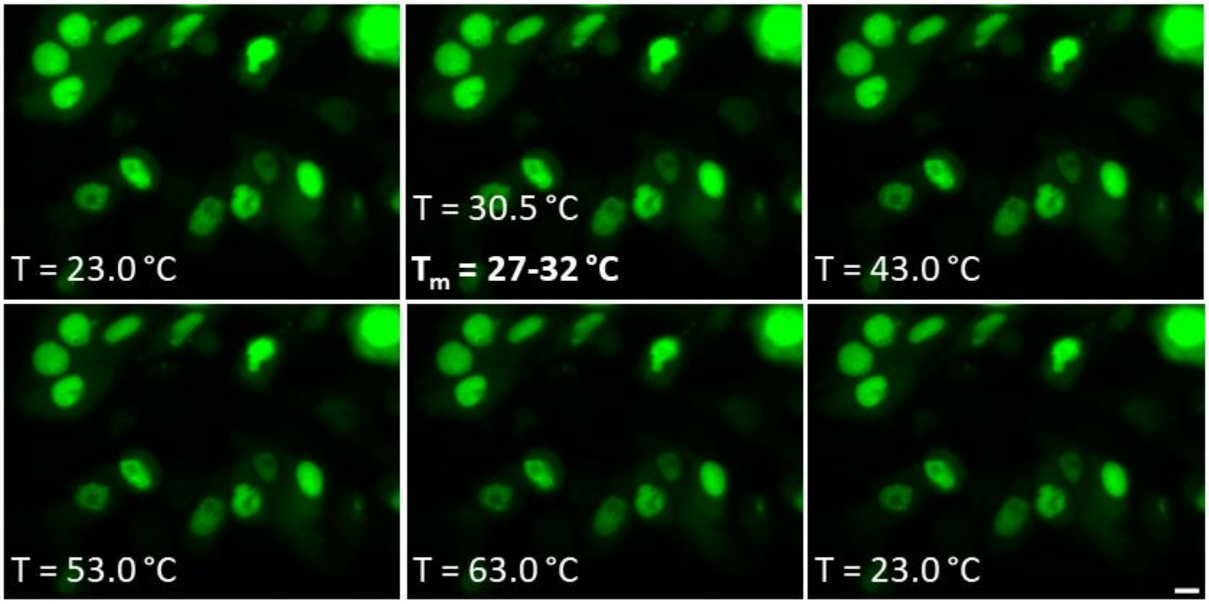

Figure S5: Acceptor fluorescence of lm4U*in HeLa cells different temperatures during the FReI experiment. lm4U* was never observed to be recruited into the nuclear speckles. Scale bar: 10 µm.

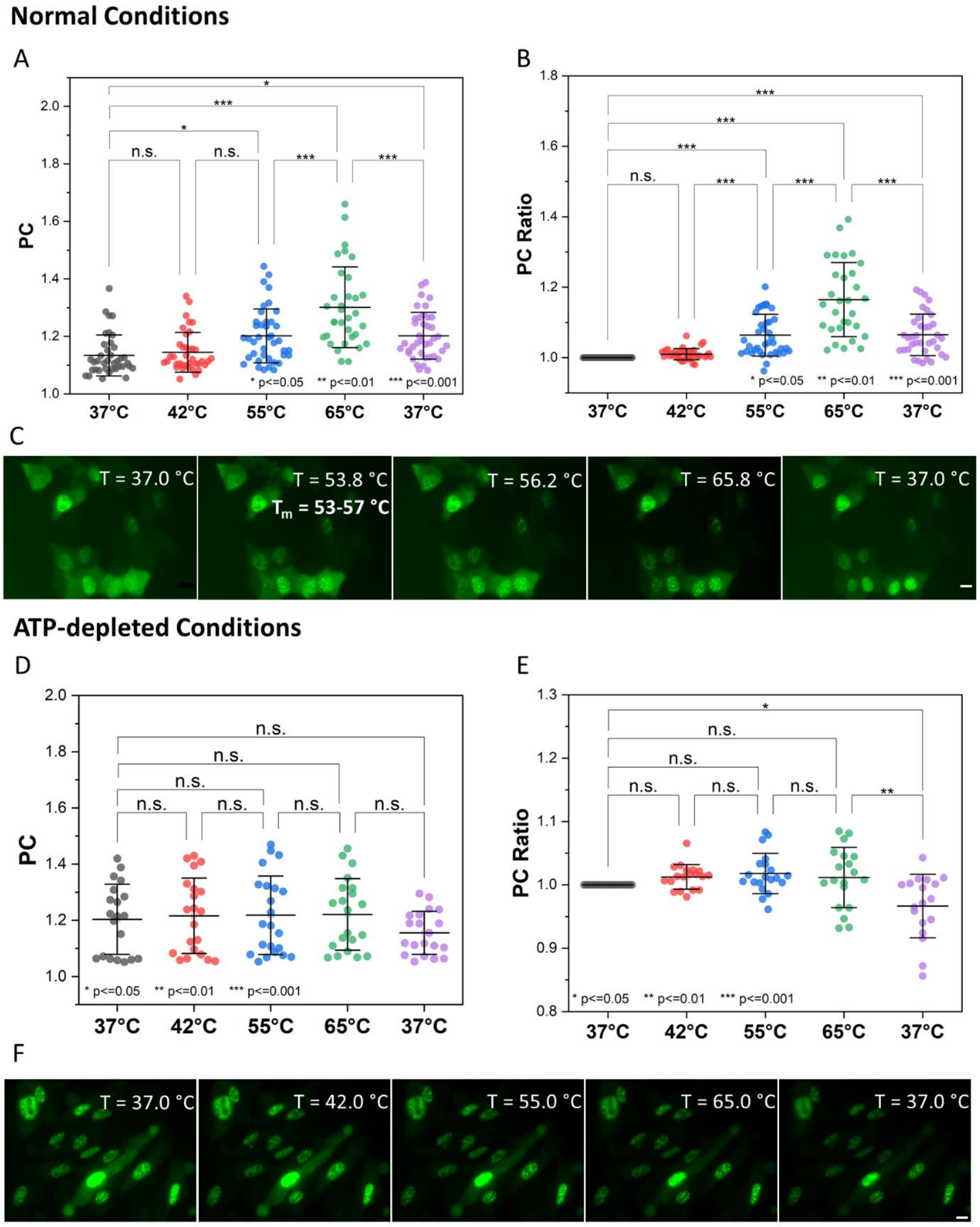

Figure S6 A, B, D, E) Local partition coefficients (PC)(A, D) and PC ratio (B, E) referenced against the initial value for $(CAG)_{20}$ in HeLa cells at different temperatures. Statistical analyses were performed by one-way ANOVA and post-hoc Tukey test (for sample sizes see Table S1). Error bars show standard deviation calculated by Gaussian error propagation (see SI materials and methods section). C, F) Acceptor fluorescence of $(CAG)_{20}$ under normal (C) and ATP-depleted conditions (F) at different temperatures during the FReI experiment. Under normal conditions, $(CAG)_{20}$

was recruited into nuclear speckles upon heating with the most significant shift at T_m. This effect was not observed under ATP- depletion. Scale bars: 10 µm.

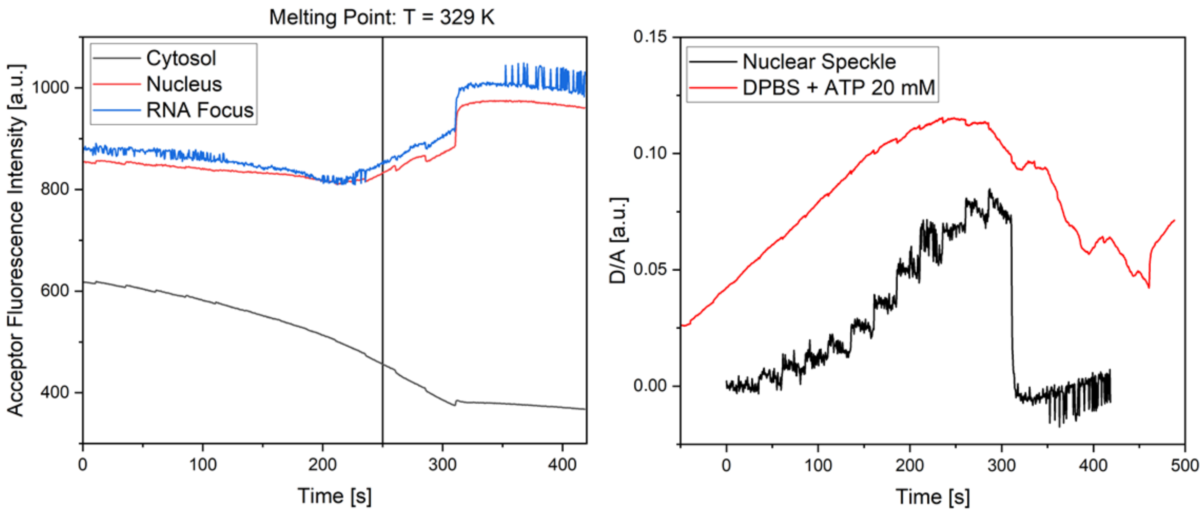

Figure S7: A) Background-corrected acceptor fluorescence intensity curves from cytosol, nucleus and a single RNA focus. An intensity increase was observed in the nucleus and foci beyond the melting temperature, suggesting the recruitment of RNA into nuclear speckles upon unfolding. B) D/A intensities as measured in a single RNA focus (black) and in DPBS supplemented with 20 mM ATP (red). At higher temperatures, the $(CAG)_{20}$ RNA in ATP showed self-association due to a non-stepwise decline of D/A, which was not observed for the RNA measured in the nuclear speckle.

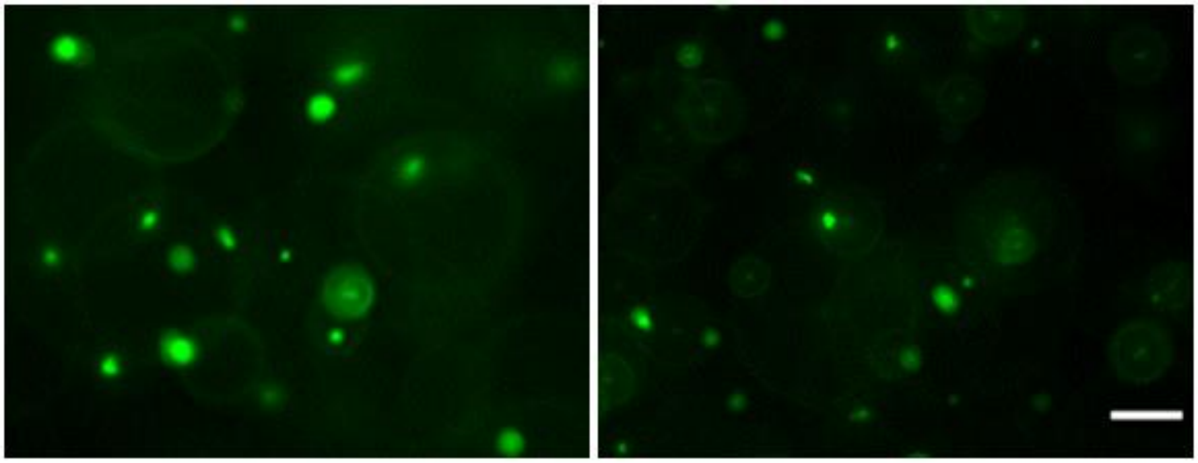

Figure S8: Aggregation of $(CAG)_{20}$ RNA at 15 (left) and 20 mM ATP (right). Scale bar: 20 µm.

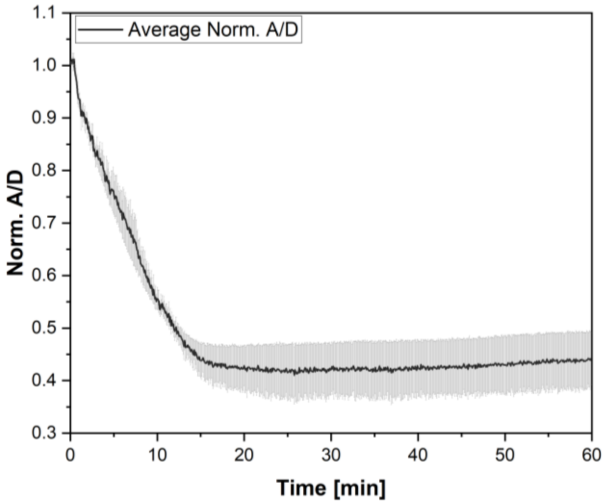

Figure S9: Relative change in ATP concentration as measured by normalized A/D fluorescence intensity (ATeam sensor) as a function of time after addition of the depletion agent (1 mM KCN and 10 mM 2-deoxyglucose; final concentration) to the measurement medium.[25] The observed ΔA/D resembles a shift in ATP concentration of 2-3 mM.[26] Values are averages (mean ± S.D.) taken from 4 individual cells.

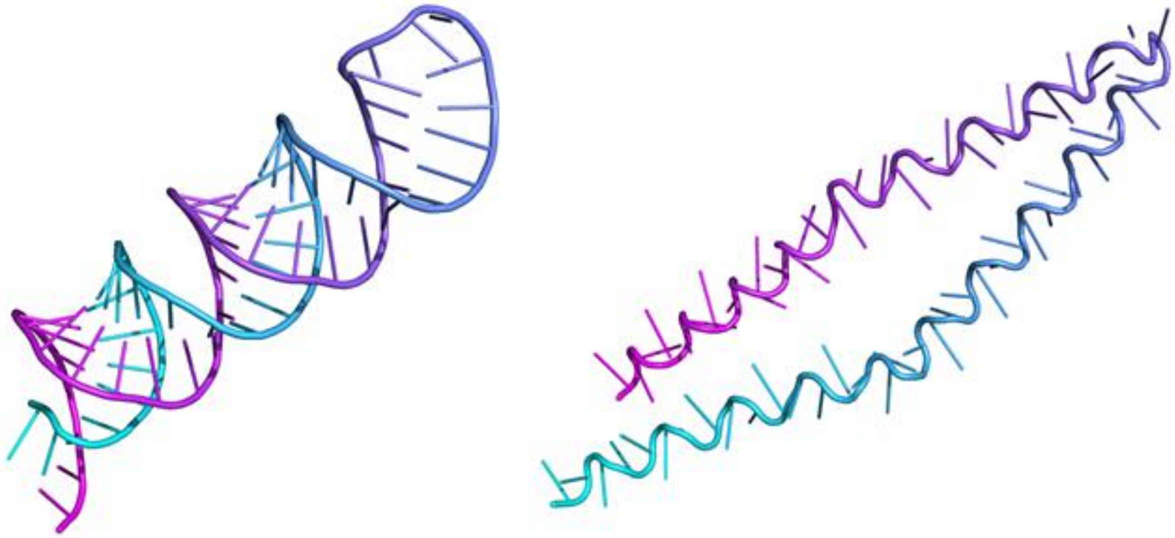

Figure S10: Native (left) and unwinded (right) conformations of $(CAG)_{20}$ RNA as used as initial structures for MD simulations.

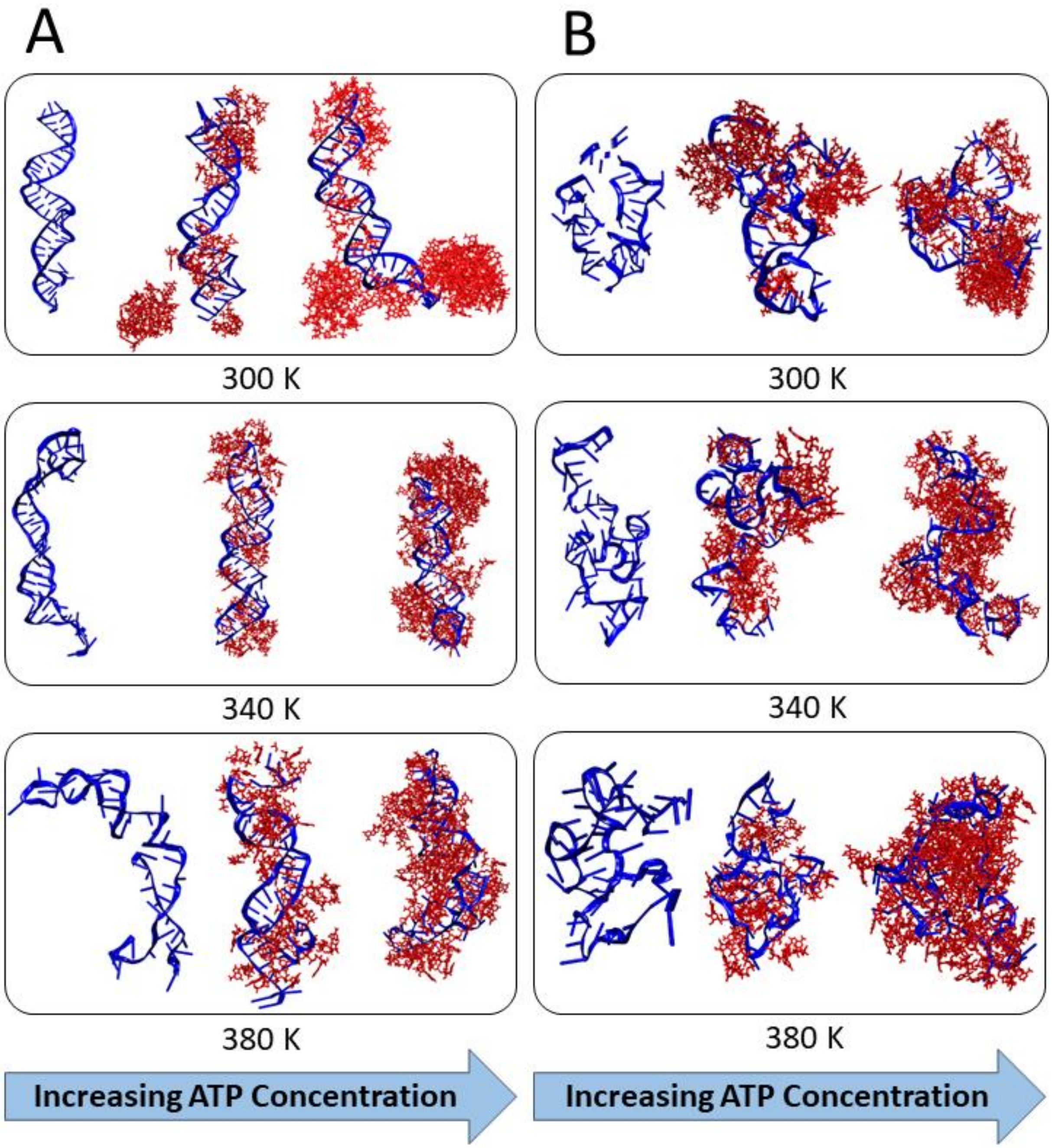

Figure S11: Final structures from MD simulations of native (A) and unwinded (B) $(CAG)_{20}$ RNA at increasing ATP concentrations (left to right). ATP concentrations were 0, 5 and 10 mM, respectively.

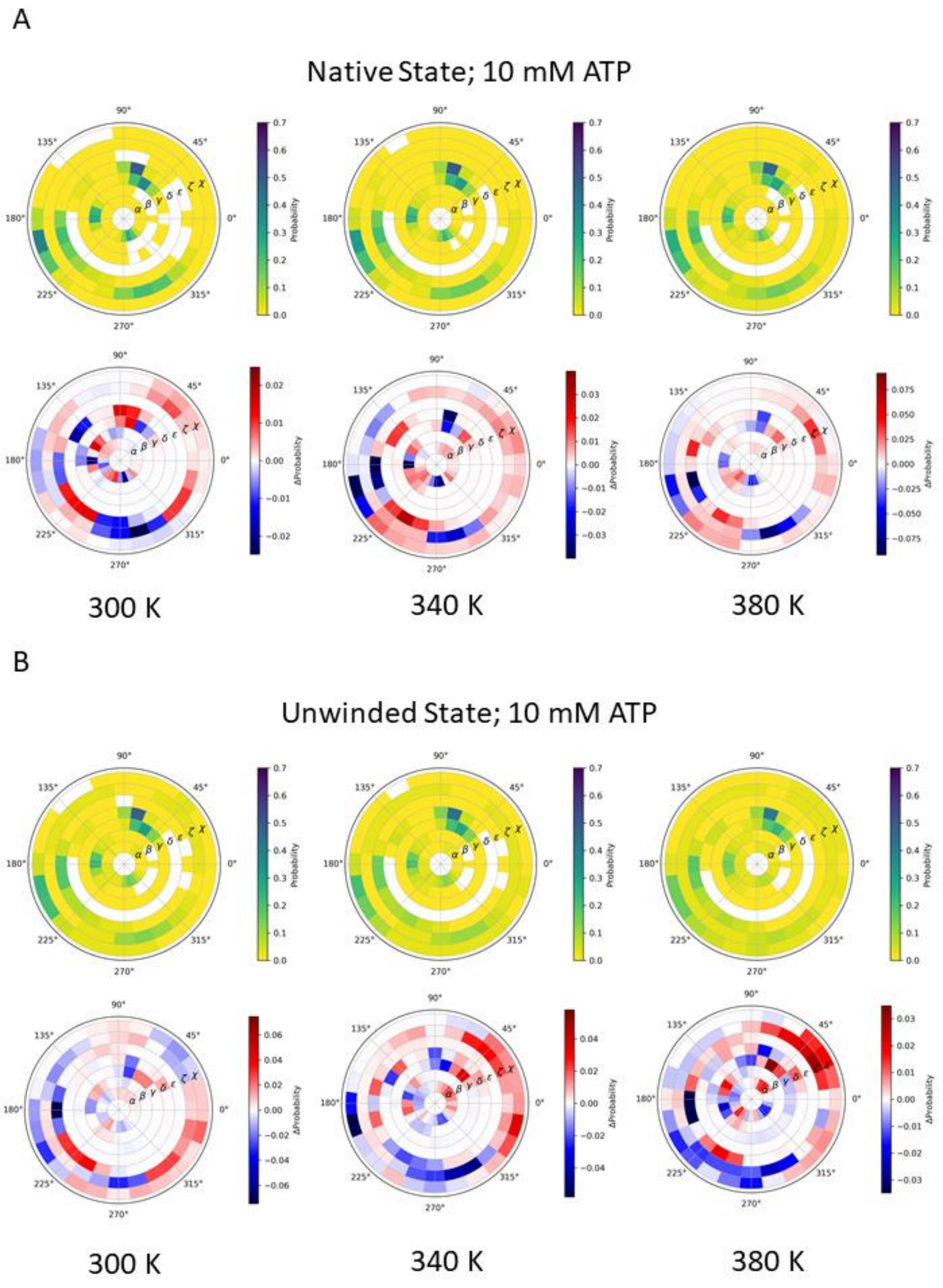
A
Native State; 10 mM ATP
Probability
ΔProbability
α β γ δ ε ζ χ
90°
45°
0°
315°
270°
225°
180°
135°
300 K
340 K
380 K
B
Unwinded State; 10 mM ATP
300 K
340 K
380 K

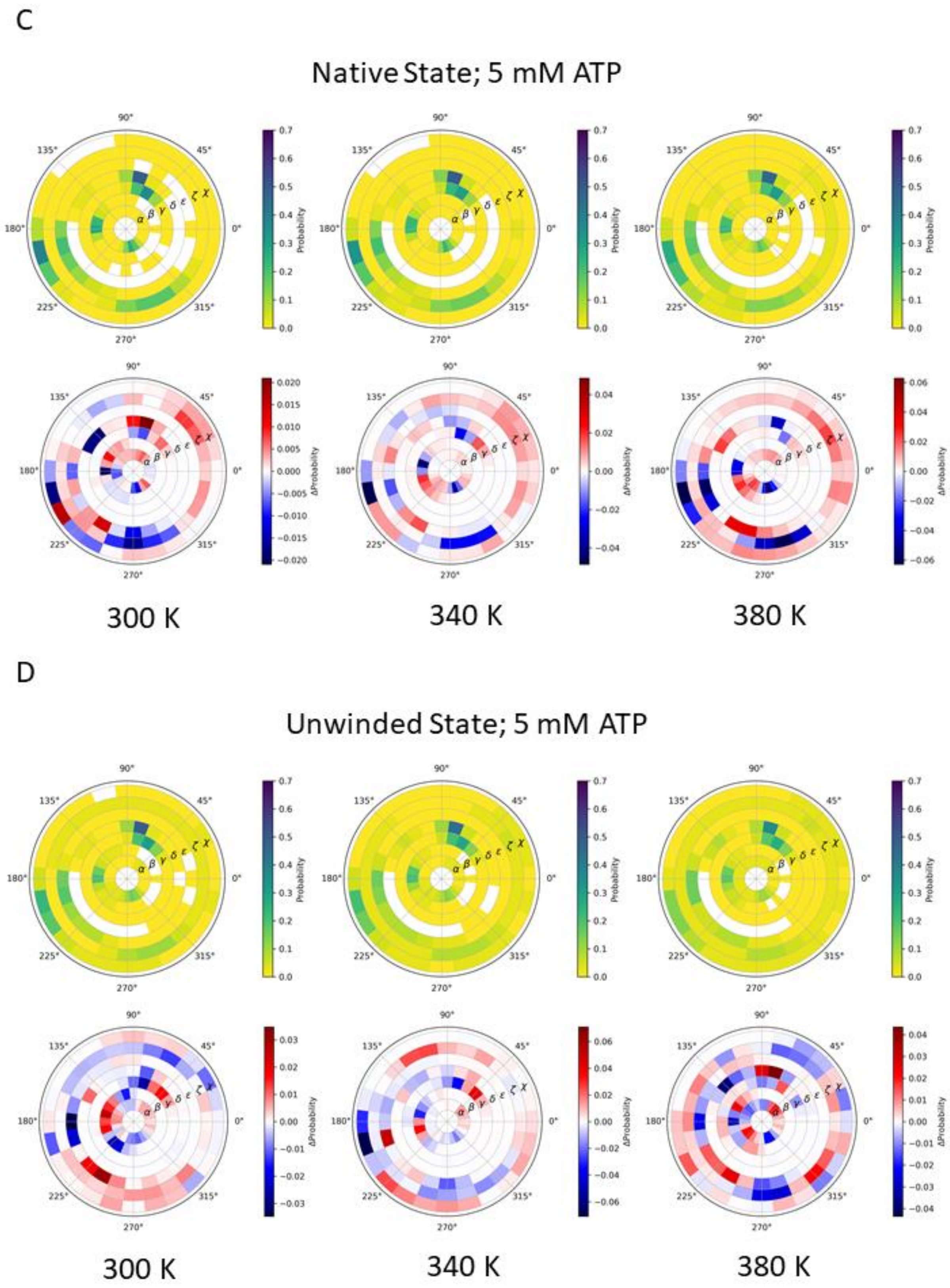

Figure S12: Torsion wheels depicting the distribution of torsion angles along $(CAG)_{20}$-RNA and the Δprobabilities calculated relative to the simulations with no ATP but otherwise same conditions for native and unwinded state at 10 (A, B) and 5 mM (C, D) of ATP.

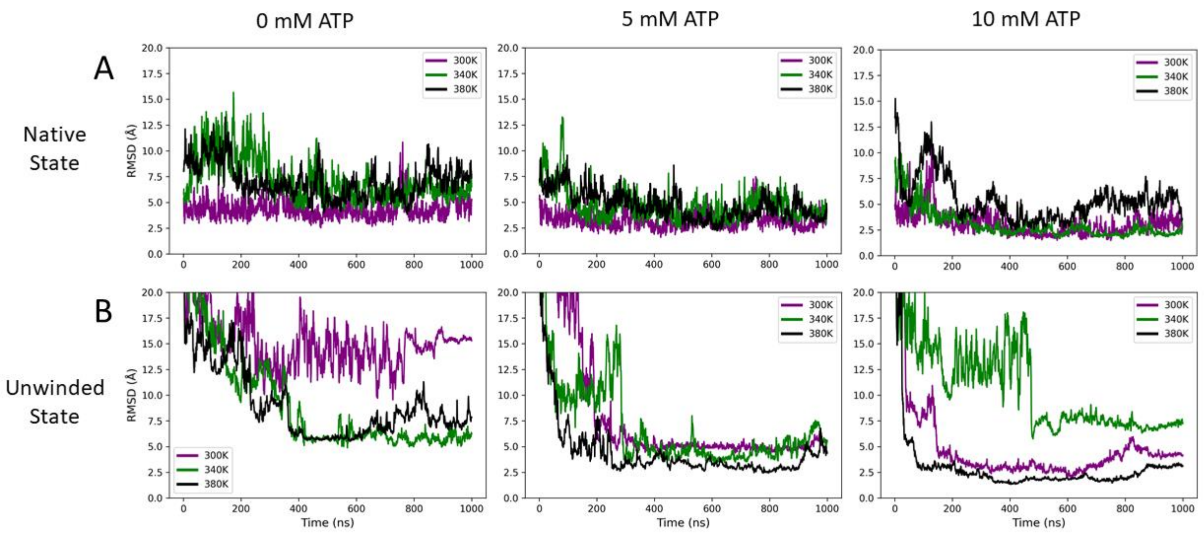

Figure S13: RMSD of native (A) and unwinded state (B) of (CAG)20 RNA between each momentaneous configuration and the average structure over the course of the simulation. As indicated, ATP, concentrations were 0, 5 and 10 mM, respectively.

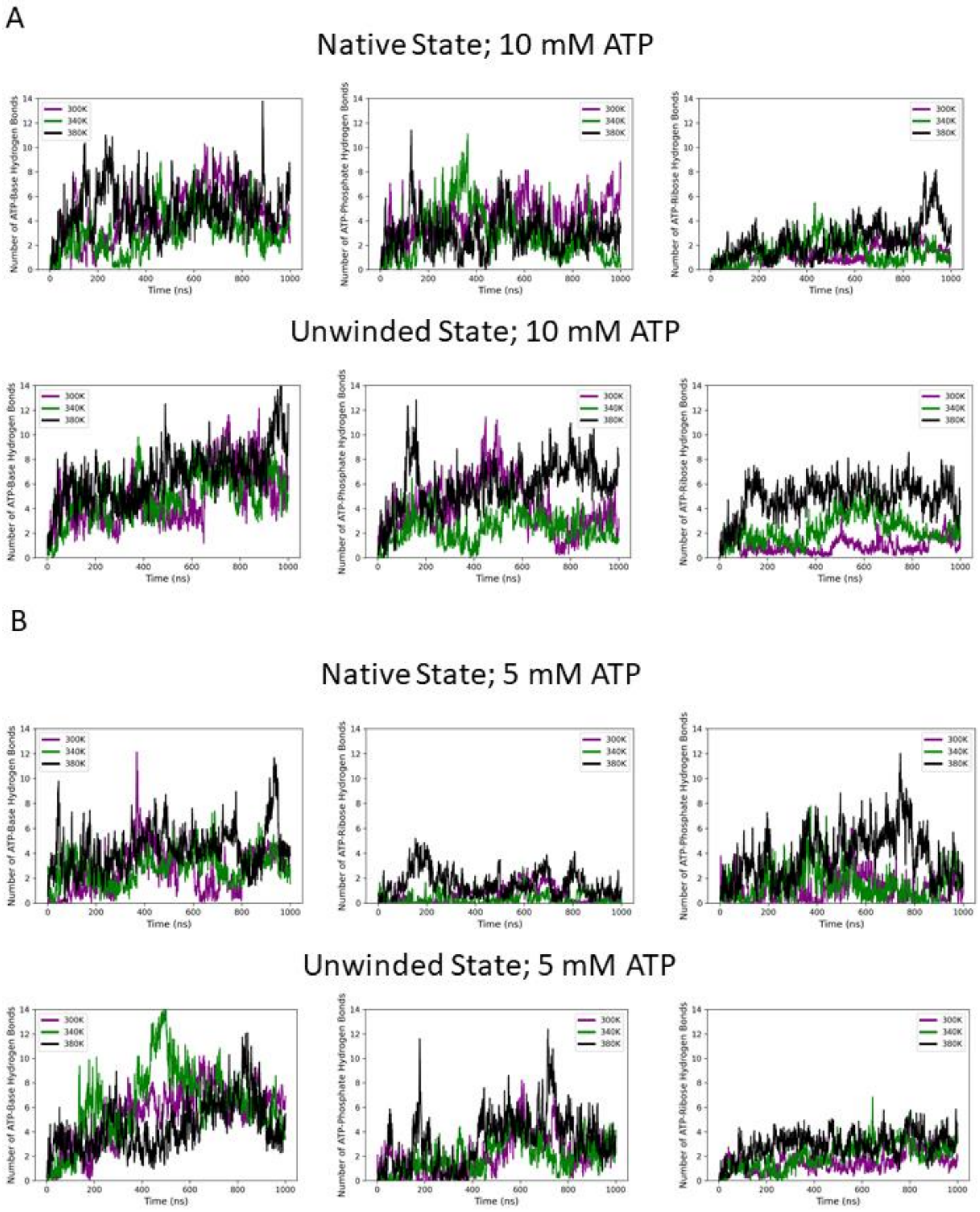

Figure S14: Number of hydrogen bonds formed between ATP and each RNA moiety at (A) 10 mM and (B) 5 mM of ATP.

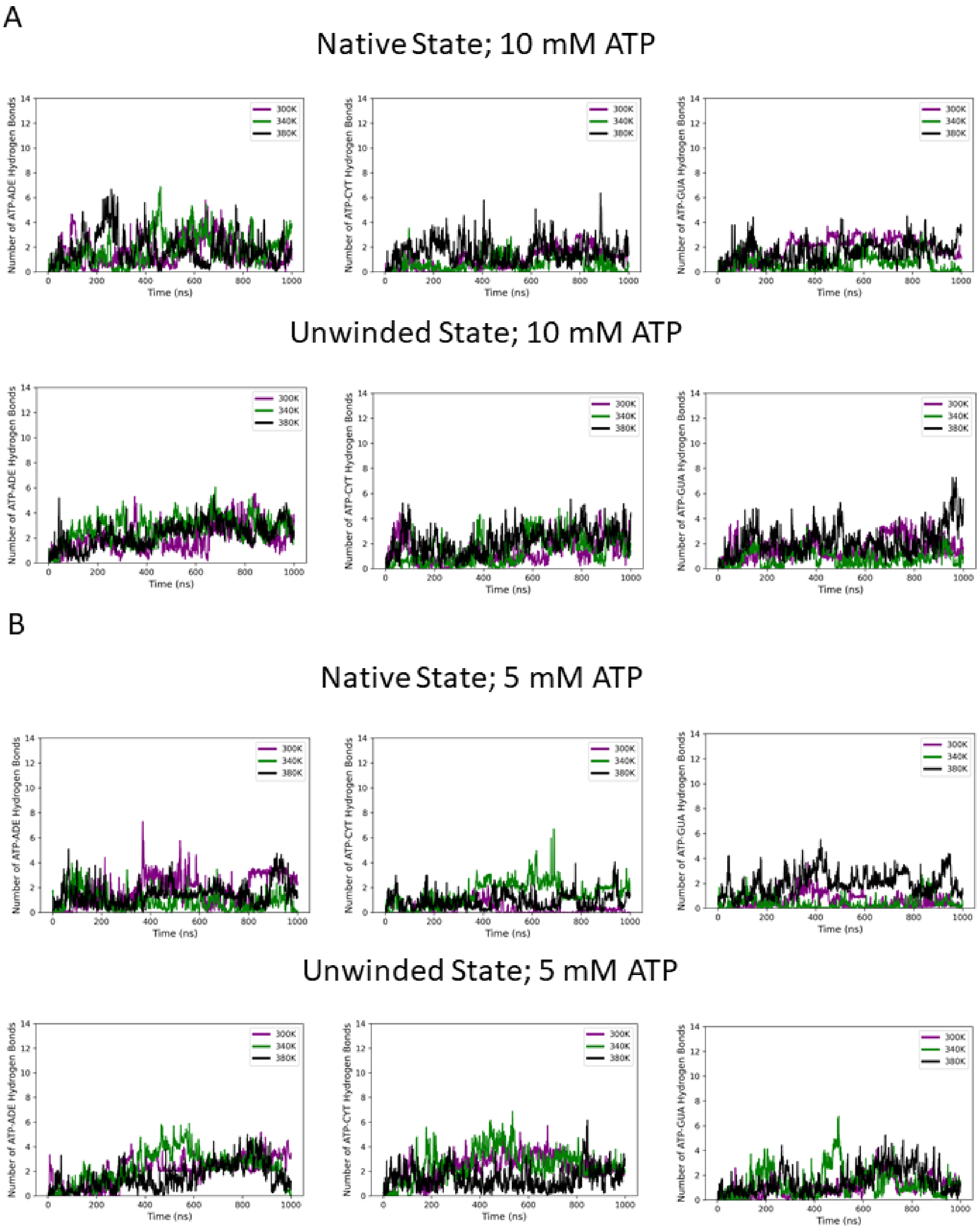

Figure S15: Number of hydrogen bonds formed between ATP and each nucleobase at (A) 10 mM and (B) 5 mM of ATP.

References

(1) Gao, M.; Gnutt, D.; Orban, A.; Appel, B.; Righetti, F.; Winter, R.; Narberhaus, F.; Müller, S.; Ebbinghaus, S. RNA Hairpin Folding in the Crowded Cell. *Angew. Chem. Int. Ed.* **2016**, *55* (9), 3224–3228.

(2) Kotera, I.; Iwasaki, T.; Imamura, H.; Noji, H.; Nagai, T. Reversible Dimerization of Aequorea victoria Fluorescent Proteins Increases the Dynamic Range of FRET-Based Indicators. *ACS Chem. Biol.* **2010**, *5* (2), 215–222.

(3) Schindelin, J.; Arganda-Carreras, I.; Frise, E.; Kaynig, V.; Longair, M.; Pietzsch, T.; Preibisch, S.; Rueden, C.; Saalfeld, S.; Schmid, B.; Tinevez, J.-Y.; White, D. J.; Hartenstein, V.; Eliceiri, K.; Tomancak, P.; Cardona, A. Fiji: an open-source platform for biological-image analysis. *Nat Methods* **2012**, *9* (7), 676–682.

(4) Carpenter, A. E.; Jones, T. R.; Lamprecht, M. R.; Clarke, C.; Kang, I. H.; Friman, O.; Guertin, D. A.; Chang, J. H.; Lindquist, R. A.; Moffat, J.; Golland, P.; Sabatini, D. M. CellProfiler: image analysis software for identifying and quantifying cell phenotypes. *Genome biology* **2006**, *7* (10), R100.

(5) Kamentsky, L.; Jones, T. R.; Fraser, A.; Bray, M.-A.; Logan, D. J.; Madden, K. L.; Ljosa, V.; Rueden, C.; Eliceiri, K. W.; Carpenter, A. E. Improved structure, function and compatibility for CellProfiler: modular high-throughput image analysis software. *Bioinformatics (Oxford, England)* **2011**, *27* (8), 1179–1180.

(6) McQuin, C.; Goodman, A.; Chernyshev, V.; Kamentsky, L.; Cimini, B. A.; Karhohs, K. W.; Doan, M.; Ding, L.; Rafelski, S. M.; Thirstrup, D.; Wiegraebe, W.; Singh, S.; Becker, T.; Caicedo, J. C.; Carpenter, A. E. CellProfiler 3.0: Next-generation image processing for biology. *PLoS biology* **2018**, *16* (7), e2005970.

(7) Stirling, D. R.; Swain-Bowden, M. J.; Lucas, A. M.; Carpenter, A. E.; Cimini, B. A.; Goodman, A. CellProfiler 4: improvements in speed, utility and usability. *BMC bioinformatics* **2021**, *22* (1), 433.

(8) Samanta, N.; Ribeiro, S. S.; Becker, M.; Laborie, E.; Pollak, R.; Timr, S.; Sterpone, F.; Ebbinghaus, S. Sequestration of Proteins in Stress Granules Relies on the In-Cell but Not the In Vitro Folding Stability. *Journal of the American Chemical Society* **2021**, *143* (47), 19909–19918.

(9) Dhar, A.; Gruebele, M. Fast Relaxation Imaging in Living Cells. *Current Protocols in Protein Science* **2011**, *65* (1), 28.1.1-28.1.19.

(10) Ebbinghaus, S.; Dhar, A.; McDonald, J. D.; Gruebele, M. Protein folding stability and dynamics imaged in a living cell. *Nat Methods* **2010**, *7* (4), 319–323.
(11) Guo, M.; Xu, Y.; Gruebele, M. Temperature dependence of protein folding kinetics in living cells. *Proceedings of the National Academy of Sciences of the United States of America* **2012**, *109* (44), 17863–17867.
(12) Fu, R.; Xu, B.; Li, D. Study of the temperature field in microchannels of a PDMS chip with embedded local heater using temperature-dependent fluorescent dye. *International Journal of Thermal Sciences* **2006**, *45* (9), 841–847.
(13) Girdhar, K.; Scott, G.; Chemla, Y. R.; Gruebele, M. Better biomolecule thermodynamics from kinetics. *The Journal of chemical physics* **2011**, *135* (1), 15102.
(14) Koulouras, G.; Panagopoulos, A.; Rapsomaniki, M. A.; Giakoumakis, N. N.; Taraviras, S.; Lygerou, Z. EasyFRAP-web: a web-based tool for the analysis of fluorescence recovery after photobleaching data. *Nucleic acids research* **2018**, *46* (W1), W467-W472.
(15) van der Spoel, D.; Lindahl, E.; Hess, B.; Groenhof, G.; Mark, A. E.; Berendsen, H. J. C. GROMACS: fast, flexible, and free. *Journal of computational chemistry* **2005**, *26* (16), 1701–1718.
(16) Lindahl; Abraham; Hess; van der Spoel. *GROMACS 2020 Manual*.
(17) Jorgensen, W. L.; Chandrasekhar, J.; Madura, J. D.; Impey, R. W.; Klein, M. L. Comparison of simple potential functions for simulating liquid water. *The Journal of chemical physics* **1983**, *79* (2), 926–935.
(18) Best, R. B.; Zhu, X.; Shim, J.; Lopes, P. E. M.; Mittal, J.; Feig, M.; Mackerell, A. D. Optimization of the additive CHARMM all-atom protein force field targeting improved sampling of the backbone φ, ψ and side-chain χ(1) and χ(2) dihedral angles. *Journal of chemical theory and computation* **2012**, *8* (9), 3257–3273.
(19) Denning, E. J.; Priyakumar, U. D.; Nilsson, L.; Mackerell, A. D. Impact of 2'-hydroxyl sampling on the conformational properties of RNA: update of the CHARMM all-atom additive force field for RNA. *Journal of computational chemistry* **2011**, *32* (9), 1929–1943.
(20) Vanommeslaeghe, K.; Hatcher, E.; Acharya, C.; Kundu, S.; Zhong, S.; Shim, J.; Darian, E.; Guvench, O.; Lopes, P.; Vorobyov, I.; Mackerell, A. D. CHARMM general force field: A force field for drug-like molecules compatible with the CHARMM all-atom additive biological force fields. *Journal of computational chemistry* **2010**, *31* (4), 671–690.

(21) Antczak, M.; Popenda, M.; Zok, T.; Sarzynska, J.; Ratajczak, T.; Tomczyk, K.; Adamiak, R. W.; Szachniuk, M. New functionality of RNAComposer: an application to shape the axis of miR160 precursor structure. *Acta biochimica Polonica* **2016**, *63* (4), 737–744.

(22) Popenda, M.; Szachniuk, M.; Antczak, M.; Purzycka, K. J.; Lukasiak, P.; Bartol, N.; Blazewicz, J.; Adamiak, R. W. Automated 3D structure composition for large RNAs. *Nucleic acids research* **2012**, *40* (14), e112.

(23) Chaudhury, S.; Lyskov, S.; Gray, J. J. PyRosetta: a script-based interface for implementing molecular modeling algorithms using Rosetta. *Bioinformatics (Oxford, England)* **2010**, *26* (5), 689–691.

(24) Reuter, J. S.; Mathews, D. H. RNAstructure: software for RNA secondary structure prediction and analysis. *BMC bioinformatics* **2010**, *11*, 129.

(25) Brylski, O.; Shrestha, P.; Gnutt, P.; Gnutt, D.; Mueller, J. W.; Ebbinghaus, S. Cellular ATP Levels Determine the Stability of a Nucleotide Kinase. *Frontiers in molecular biosciences* **2021**, *8*, 790304.

(26) Lerchundi, R.; Huang, N.; Rose, C. R. Quantitative Imaging of Changes in Astrocytic and Neuronal Adenosine Triphosphate Using Two Different Variants of ATeam. *Frontiers in cellular neuroscience* **2020**, *14*, 80.
